# Supplementary material for: Metal–peptide rings form highly entangled topologically inequivalent frameworks with the same ring- and crossing-numbers
Source: Nat Commun. 2019 Feb 22;10:921. doi: 10.1038/s41467-019-08879-7 (PMC6384881; doi:10.1038/s41467-019-08879-7)
Supplement: Supplementary file 1 — Supplementary Information [file 41467_2019_8879_MOESM1_ESM.pdf]

Supplementary Information

Metal–peptide rings form highly entangled topologically inequivalent frameworks with the same ring- and crossing-numbers

Sawada et al.

Supplementary Methods

Synthesis of ligands **3**, **5**, **7** and **9** was carried out according to the following procedure:

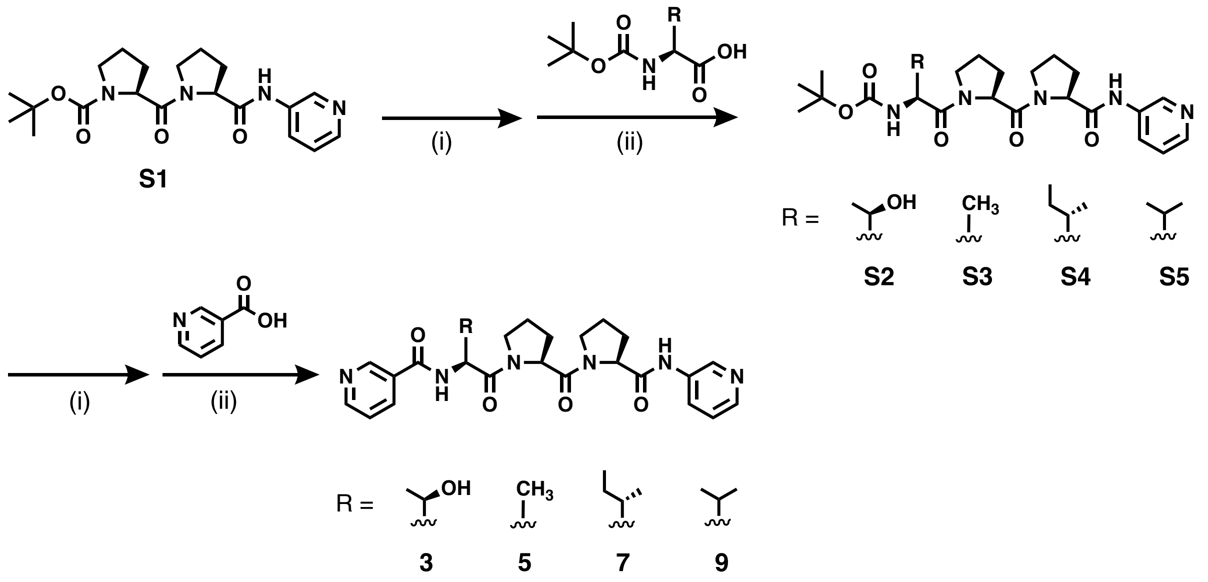

(i) HCl in MeOH / 1,4-dioxane, r.t., 1–1.5 h, (ii) HOBt•H_2O, EDCI•HCl, DIEA in $CHCl_3$, r.t., 1–2 d.

<u>*N*-(3-Pyridyl)-boc-prolylprolineamide (**S1**)</u>

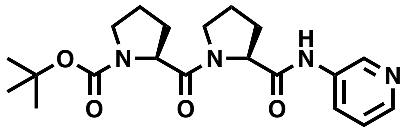

Compound **S1** was prepared according to the previous report.[1]

<u>*N*-(3-Pyridyl)-boc-threonylprolylprolineamide (**S2**)</u>

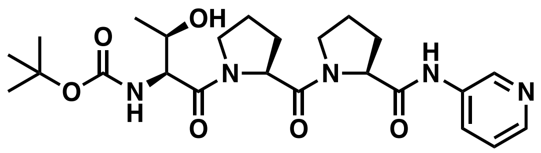

S1 2.9 g (7.5 mmol) was dissolved in methanol (20 mL). To the solution, 4N HCl (in 1,4-dioxane) 9.3 mL (37 mmol) was slowly added. After the mixture was stirred for 1 h, the solvents were removed by evaporation. The resulting pale yellow solid was dried *in vacuo*. Quantitative removal of the boc group was confirmed by ^{1}H NMR measurement.

The obtained *N*-(3-pyridyl)-prolylprolineamide dihydrochloride 2.7 g (7.5 mmol), Boc-L-Thr-OH 1.8 g (8.2 mmol), EDCI•HCl 1.7 g (9.0 mmol), HOBt•H_2O 1.4 g (9.0 mmol) and DIEA 4.5 mL (7.5 mmol) were dissolved in CH_2Cl_2 (50 mL). After the mixture was stirred overnight at ambient temperature, the solution was washed with sat. $NaHCO_3$ aq. (20 mL, twice) and brine (20 mL, once), and then the organic layer was dried over $MgSO_4$. The crude product solution was concentrated by evaporation, and then applied to SiO_2 column chromatography ($CHCl_3$: MeOH = 100 : 0 → 90 : 10). The fractions were collected and concentrated by evaporation and dried *in vacuo*. The white solid 1.6 g of **S2** was obtained (44% yield).

m.p.: 117–125 °C; **ESI-MS** (*m/z*): $[M+Na]^+$ calcd for $C_{24}H_{35}N_5O_6Na$: 512.3, found: 512.3; **analysis**: (% calcd, % found for $C_{24}H_{35}N_5O_6$•H_2O): C (56.79, 56.83), H (7.35, 7.30), N (13.80, 13.74); **^{1}H-NMR** (500 MHz, $CDCl_3$, 300 K): δ 9.50 (s, 1H, N*H*-Py), 8.54 (s, 1H, PyH_2), 8.31 (d, J = 4.0 Hz, 1H, PyH_6), 8.06 (d, J = 8.0 Hz, 1H, PyH_4), 7.23 (dd, J = 8.0 Hz, 5.0 Hz, 1H, PyH_5), 5.45 (d, J = 9.0 Hz, 1H, ThrN*H*), 4.80 (dd, J = 8.0 Hz, 1.5 Hz, 1H, ProH_α), 4.69 (dd, J = 8 Hz, 6 Hz, 1H, ProH_α), 4.45 (d, J = 8.0 Hz, 1H, ThrH_α), 4.26 (m, 1H, ThrH_β), 3.88–3.75, 3.63, 3.58 (m, 2H, ProH_δ), 2.72–1.81 (m, 8H, ProH_β, ProH_γ), 1.44 (s, 9H, *t*-Bu), 1.24 (d, J = 6.0 Hz, 3H, ThrH_γ), *conformer*: 9.64 (s, N*H*-Py), 9.06 (s, PyH_2), 8.37 (d, PyH_6), 8.15 (d, PyH_4), 4.48 (m, ProH_α), 4.28 (m, ThrH_β), 3.50, 3.58 (brs, ProH_δ), 1.41 (s, *t*-Bu), 1.26 (d, ThrH_γ); **^{13}C-NMR** (125 MHz, $CDCl_3$, 300 K): δ 173.3, 171.2, (*C*O), 169.4 (Pro*C*O), 156.1 (Boc*C*O), 145.1 (PyC_6), 141.3 (PyC_2), 126.8 (PyC_4), 123.6 (PyC_5), 79.9 (BocC_q), 67.3 (ThrC_β), 60.8 (ProC_α), 58.3 (ProC_α), 56.1 (ThrC_α), 47.6 (ProC_δ), 28.7, 28.3 (ProC_β), 28.3 (BocCH_3), 26.2, 25.3 (ProC_γ), 18.5 (ThrC_γ), *conformer*: 170.6, 169.5, 145.5, 142.5, 127.6, 123.2, 80.0, 67.1, 61.7, 59.7, 56.4, 47.0, 47.1, 32.0, 29.7, 28.2, 25.6, 22.1, 18.6.

N-(3-Pyridyl)-boc-alanylprolylprolineamide (**S3**)

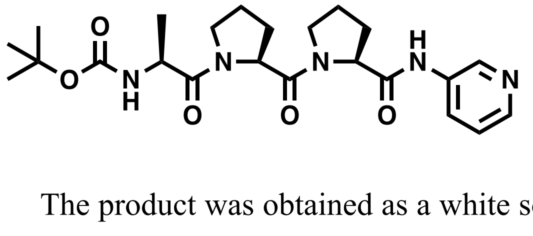

The product was obtained as a white solid 1.7 g (91% yield) following the same procedure for **S2**.

m.p.: 89.0–93.1 °C; **HR-MS** (*m/z*): $[M+H]^+$ calcd for $C_{23}H_{34}N_5O_5$: 460.2554, found: 460.2596; ^{1}**H-NMR** (500 MHz, $CDCl_3$, 300 K): δ 9.96 (s, 1H, N*H*-Py), 8.46 (s, 1H, PyH_2), 8.16 (s, 1H, PyH_6), 7.95 (d, J = 8.5 Hz, 1H, PyH_4), 7.04 (dd, J = 8.0 Hz, 5.0 Hz, 1H, PyH_5), 5.40 (d, J = 8.5 Hz, 1H, AlaN*H*), 4.74–4.70 (m, 2H, ProH_α), 4.49 (m, 1H, AlaH_α), 3.84 (m, 1H, ProH_δ), 3.76 (m, 1H, ProH_δ), 3.64 (m, 2H, ProH_δ), 2.18, 2.03 (m, 4H, ProH_γ), 2.27, 2.18, 2.07 (m, 4H, ProH_β), 1.43 (s, 9H, *t*-Bu), 1.30 (d, J = 7 Hz, 3H, AlaH_β), *conformer*: 9.84 (s, N*H*-Py), 9.08 (s, PyH_2), 8.35 (s, PyH_6), 8.23 (d, PyH_4), 7.23 (dd, PyH_5), 5.37 (d, AlaN*H*), 4.44 (m, AlaH_α), 2.67 (s, ProH_β); ^{13}**C-NMR** (125 MHz, $CDCl_3$, 300 K): δ 171.6 (Ala*C*O), 171.5 (Pro*C*O), 170.5 (Pro*C*O), 155.1 (Boc*C*O), 144.4 (PyC_6), 140.8 (PyC_2), 135.3 (PyC_3), 126.2 (PyC_4), 123.3 (PyC_5), 79.6 (BocC_q), 60.8 (ProC_α), 58.0 (ProC_α), 47.7 (AlaC_α), 47.5 (ProC_δ), 47.2 (ProC_δ), 28.3 (BocCH_3), 28.4, 28.1 (ProC_β), 25.1, 25.0 (ProC_γ), 18.3 (AlaC_β), *conformer*: 172.0, 170.8, 169.8, 155.3, 145.2, 142.5, 134.9, 127.5, 123.2, 79.8, 61.7, 59.1, 47.7, 47.4, 46.8, 31.8, 28.3, 28.2, 25.4, 22.2, 17.6.

N-(3-Pyridyl)-boc-isoleucylprolylprolineamide (**S4**)

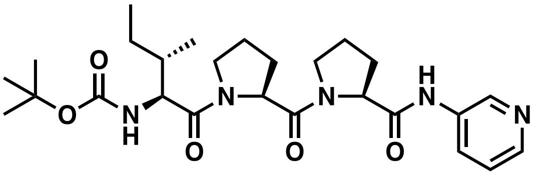

The product was obtained as a white solid 0.42 g (84% yield) following the same procedure for **S2**.

m.p.: 181–182 °C; **HRMS** (*m/z*): $[M+H]^+$ calcd for $C_{26}H_{40}N_5O_5$, 502.3024; found, 502.3028; ^{1}**H-NMR** (500 MHz, $CDCl_3$, 300 K): δ 10.07 (s, 1H, N*H*-Py), 8.46 (s, 1H, PyH_2), 8.15 (d, J = 4.5 Hz, 1H, PyH_6), 7.93 (d, J = 8.5 Hz, 1H, PyH_4), 7.02 (dd, J = 8.5 Hz, 5.0 Hz, 1H, PyH_5), 5.29 (d, J = 9.5 Hz, 1H, IleN*H*), 4.78 (dd, J = 8.0 Hz, 5.0 Hz, 1H, Pro(2)H_α), 4.72 (t, J = 6.0 Hz, 1H, Pro(3)H_α), 4.30 (t, J = 9.0 Hz, 1H, IleH_α), 3.89 (m, 2H, ProH_δ), 3.71 (m, 2H, ProH_δ), 2.29, 2.18, 2.11, 2.04 (m, 8H, ProH_β, ProH_γ), 1.75 (m, 1H, IleH_β), 1.60, 1.17 (m, 2H, IleH_γ), 1.43 (s, 9H, *t*-Bu), 0.98 (d, J = 7.0 Hz, 3H, Ile$H_{\gamma'}$), 0.85 (t, J = 7.5 Hz, 3H, IleH_δ), *conformer*: 10.05 (s, N*H*-Py), 9.08 (s, PyH_2), 8.34 (d, PyH_4), 8.30 (d, PyH_6), 7.25 (dd, PyH_5), 5.42 (d, IleN*H*), 4.49 (d, Pro(2)H_α), 4.41 (t, Pro(3)H_α), 4.23 (t, IleH_α), 4.00, 3.64 (m, ProH_δ), 1.08 (d, Ile$H_{\gamma'}$), 0.93 (t, IleH_δ); ^{13}**C-NMR** (125 MHz, $CDCl_3$, 300 K): δ 171.4 (*C*O), 171.3 (*C*O), 170.6 (Pro(3)*C*O), 155.5 (Boc*C*O), 144.1 (PyC_6), 140.6 (PyC_2), 135.4 (PyC_3), 126.1 (PyC_4), 123.1

(PyC_5), 79.4 (BocC_q), 60.7 (ProC_α), 58.2 (ProC_α), 56.0 (IleC_α), 47.7, 47.5 (ProC_δ), 37.9 (IleC_β), 28.4, 28.2, 28.1 (ProC_β, BocCH_3), 25.0, 24.9 (ProC_γ), 24.4 (IleC_γ), 15.1 (Ile$C_{\gamma'}$), 10.9 (IleC_δ), *conformer*: 171.7, 170.6, 170.0, 155.7, 144.9, 142.3, 135.0 127.4, 79.4, 61.7, 59.2, 56.2, 46.8, 37.3, 31.8, 25.4, 22.2, 18.3, 15.0, 10.8.

N-(3-Pyridyl)-boc-valylprolylprolineamide (**S5**)

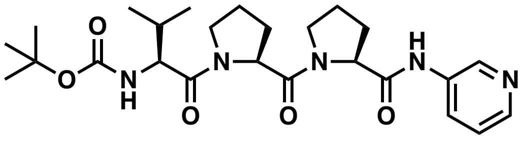

The product was obtained as a white solid 0.70 g (72% yield) following the same procedure for **S2**.

m.p.: 119–120 °C; **ESI-MS** (*m/z*): $[M+Na]^+$ calcd for $C_{25}H_{37}N_5O_5Na$, 510.3; found, 510.3; **analysis** (% calcd, % found for $C_{25}H_{37}N_5O_5{\bullet}(H_2O)_{0.5}$): C (60.47, 60.28), H (7.71, 7.83), N (14.10, 13.99); **^{1}H-NMR** (500 MHz, DMSO-d_6, 300 K): δ 10.25 (s, 1H, N*H*-Py), 8.71 (d, J = 2.5 Hz, 1H, PyH_2), 8.25 (dd, J = 4.5 Hz, 1.5 Hz, 1H, PyH_6), 8.02 (m, 1H, PyH_4), 7.33 (dd, J = 8.5 Hz, 5.0 Hz, 1H, PyH_5), 6.73 (d, J = 8.5 Hz, 1H, IleN*H*), 4.62 (dd, J = 9.0 Hz, 5.0 Hz, 1H, Pro(2)H_α), 4.44 (dd, J = 8.0 Hz, 4.0 Hz, 1H, Pro(3)H_α), 3.99 (t, J = 8.0 Hz, 1H, ValH_α), 3.73, 3.61, 3.51 (m, 4H, ProH_δ), 2.18 (m, 2H, ProH_β), 2.25–1.77 (m, 7H, ProH_β, ProH_γ, ValH_β), 1.37 (s, 9H, *t*-Bu), 0.89, 0.84 (d, J = 7.0 Hz, 6H, ValH_γ), (*an additional conformer exists*); **^{13}C-NMR** (500 MHz, DMSO-d_6, 300 K): δ 171.6 (Pro(3)*C*O), 170.4, 170.4 (Pro(2)*C*O, Val*C*O), 156.0 (Boc*C*O), 144.6 (PyC_6), 141.0 (PyC_2), 136.2 (PyC_3), 126.3 (PyC_4), 124.1 (PyC_5), 78.4 (BocC_q), 60.4 (Pro(3)C_α), 58.0 (Pro(2)C_α), 57.6 (ValC_α), 47.5, 47.2 (ProC_δ), 30.4 (ValC_β), 29.7 (ProC_γ), 28.7 (BocCH_3), 28.4 (ProC_β), 25.1, 25.0 (ProC_γ), 19.5, 18.9 (ValC_γ), (*an additional conformer exists*).

Tripeptide ligand (**3**)

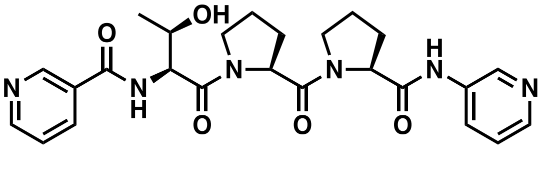

1.6 g (3.3 mmol) of **S2** was dissolved in methanol (20 mL). To the solution, 4N HCl (in 1,4-dioxane) 4.0 mL (16 mmol) was slowly added. After the mixture was stirred for 1 h, the

solvents were removed by evaporation. The resulting white solid was dried *in vacuo*. Quantitative removal of the boc group was confirmed by ^{1}H NMR measurement.

The obtained *N*-(3-pyridyl)-threonylprolylprolineamide dihydrochloride 1.5 g (3.3 mmol), nicotinic acid 0.44 g (3.6 mmol), EDCI•HCl 0.76 g (3.9 mmol), HOBt•H_2O 0.60 g (3.9 mmol) and DIEA 2.0 mL (11 mmol) were dissolved in $CHCl_3$ (50 mL). After the mixture was stirred for 2 days at ambient temperature, the solution was concentrated by evaporation, and then applied to SiO_2 column chromatography (EtOAc : MeOH = 9 : 1 → 1 : 1). The fractions were concentrated by evaporation. The material was further purified by using a preparative SEC (W252 column, eluent: MeOH : H_2O = 95 : 5 (*v/v*)). The resulting white solid was dissolved in *ca.* 30 mL of H_2O, and then freeze-dried. White powder 0.68 g of **3** was obtained (45% yield).

m.p.: 127–130 ºC; **HRMS** (*m/z*): $[M+H]^+$ calcd for $C_{25}H_{31}N_6O_5$, 495.2350; found, 495.2377; **analysis**: (% calcd, % found for $C_{25}H_{30}N_6O_5$•$(H_2O)_{1.5}$): C (57.57, 57.61), H (6.38, 6.33), N (16.11, 16.24); **^{1}H-NMR** (500 MHz, $CDCl_3$, 300 K): δ 9.54 (s, 1H, N*H*-Py), 9.08 (d, *J* = 2.0 Hz, 1H, PyH_{2N}), 8.73 (dd, *J* = 5.0 Hz, 2.0 Hz, 1H, PyH_{6N}), 8.54 (d, *J* = 2.0 Hz, 1H, PyH_{2C}), 8.31 (dd, *J* = 5.0 Hz, 1.5 Hz, 1H, PyH_{6C}), 8.12 (ddd, 1H, *J* = 8.0 Hz, 2.0 Hz, 2.0 Hz, PyH_{4N}), 8.08 (ddd, *J* = 8.0 Hz, 2.5 Hz, 1.5 Hz, 1H, PyH_{4C}), 7.37 (ddd, *J* = 8.0 Hz, 5.0 Hz, 1.0 Hz, 2H, PyH_{5N}), 7.33 (d, *J* = 8.5 Hz, 1H, ThrN*H*), 7.22 (dd, *J* = 8.5 Hz, 4.5 Hz, 1H, PyH_{5C}), 5.01 (dd, *J* = 8.5 Hz, 2.0 Hz, 1H, ThrNH_α), 4.79 (dd, *J* = 8.0 Hz, 2.0 Hz, 2H, ProH_α), 4.74 (dd, *J* = 8.0 Hz, 6.5 Hz, 2H, ProH_α), 4.43 (m, 1H, ThrH_β), 4.00–3.58 (m, 5H, ProH_δ, OH), 2.75–1.88 (m, 8H, ProH_β, ProH_γ), 1.30 (d, *J* = 6.5 Hz, 3H, ThrH_γ), (*additional two sets of conformers exist*); **^{13}C-NMR** (125 MHz, $CDCl_3$, 300 K): δ 172.8 (Pro*C*O), 169.9 (Thr*C*O), 169.5 (Pro*C*O), 165.8 (Py*C*O), 152.6 (PyC_{6N}), 148.5 (PyC_{2N}), 145.1 (PyC_{6C}), 141.2 (PyC_{2C}), 135.0 (PyC_{4N}), 135.0 (PyC_{3C}), 129.4 (PyC_{3N}), 126.8 (PyC_{4C}), 123.5 (PyC_{5N}, PyC_{5C}), 67.0 (ThrC_β), 60.9, 58.6 (ProC_α), 55.9 (ThrC_α), 58.6 (ThrC_β), 55.9 (ThrC_β), 47.8, 47.5 (ProC_δ), 28.6, 26.7, 25.4, 25.3 (ProC_β, ProC_γ), 19.1 (ThrC_γ), (*additional two sets of conformers exist*).

<u>Tripeptide ligand (**5**)</u>

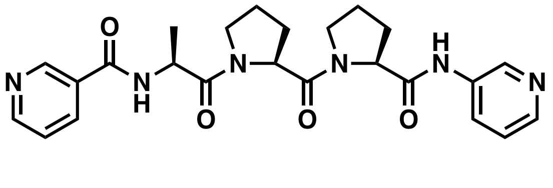

The product was obtained as a white solid 0.74 g (59% yield) following the same procedure for **3** and purified by column chromatography (EtOAc : MeOH = 10 : 1 → 2 : 1). The material was further purified by using a preparative SEC (W252 column, eluent: MeOH : H_2O = 95 : 5 (*v/v*)).

m.p.: 130–137 °C; **HRMS**: $[M+Na]^+$ calcd for $C_{24}H_{28}N_6O_4Na$: 487.2064; found: 487.2024; **analysis**: (% calcd, % found for $C_{24}H_{28}N_6O_4{\bullet}(H_2O)_{0.5}$): C (60.88, 60.74), H (6.17, 6.19), N (17.75, 17.99); **^{1}H-NMR** (500 MHz, DMSO-d_6, 300 K): δ 10.25 (s, 1H, N*H*-Py), 9.03 (d, J = 1.5 Hz, 1H, PyH_{2N}), 8.78 (d, J = 7.0 Hz, 1H, AlaN*H*), 8.73 (d, J = 2.0 Hz, 1H, PyH_{2C}), 8.71 (dd, J = 5.0 Hz, 2.0 Hz, 1H, PyH_{6N}), 8.26 (dd, J = 4.5 Hz, 1.5 Hz, 1H, PyH_{6C}), 8.22 (ddd, J = 8.0 Hz, 2.0 Hz, 1.5 Hz, 1H, PyH_{4N}), 8.03 (ddd, J = 8.5 Hz, 2.5 Hz, 1.5 Hz, 1H, PyH_{4C}), 7.51 (dd, J = 8.0 Hz, 5.0 Hz, 1H, PyH_{5N}), 7.34 (dd, J = 8.5 Hz, 4.5 Hz, 1H, PyH_{5C}), 4.72 (m, 1H, AlaH_α), 4.64 (dd, J = 8.5 Hz, 4.5 Hz, 1H, Pro(2)H_α), 4.44 (dd, J = 8.5 Hz, 4.5 Hz, 1H, Pro(3)H_α), 3.76, 3.54 (m, 2H, Pro(2)H_δ), 3.72, 3.50 (m, 2H, Pro(3)H_δ), 2.19, 1.85 (m, 2H, Pro(2)H_β), 2.15, 1.91 (m, 2H, Pro(3)H_β), 1.94, 1.93 (m, 2H, Pro(2)H_γ), 2.01, 1.92 (m, 2H, Pro(3)H_γ), 1.31 (d, J = 7.5 Hz, 3H, AlaH_α), (*additional two sets of conformers exist*); **^{13}C-NMR** (125 MHz, DMSO-d_6, 300 K): δ 171.6 (Pro(3)*C*O), 170.6 (Ala*C*O), 170.4 (Pro(2)*C*O), 165.0 (Py*C*O), 152.4 (PyC_{6N}), 149.0 (PyC_{2N}), 144.6 (PyC_{6C}), 141.1 (PyC_{2C}), 136.1 (PyC_{3C}), 135.6 (PyC_{4N}), 129.8 (PyC_{3N}), 126.4 (PyC_{4C}), 124.1 (PyC_{5C}), 123.8 (PyC_{5N}), 60.5 (Pro(3)C_α), 58.0 (Pro(2)C_α), 47.5 (AlaC_α), 47.1 (Pro(2)C_δ), Pro(3)C_δ), 29.7 (Pro(3)C_β), 28.3 (Pro(2)C_β), 25.2 (Pro(3)C_γ), 25.0 (Pro(2)C_γ), 16.8 (AlaC_α), (*additional two sets of conformers exist*).

Tripeptide ligand (**7**)

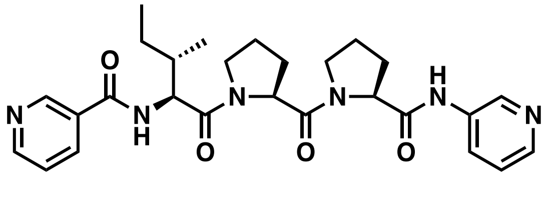

The product was obtained as a white solid 0.14 g (32% yield) following the same procedure for **3** and purified by column chromatography (EtOAc : MeOH = 10 : 1 → 2 : 1). The material was further purified by using a preparative SEC (W252 column, eluent: MeOH).

m.p.: 122–131 °C; **HRMS**: $[M+H]^+$ calcd for $C_{27}H_{35}N_6O_4$: 507.2705, found: 507.2714; **^{1}H-NMR** (500 MHz, DMSO-d_6, 300 K): δ 10.26 (s, 1H, N*H*-Py), 9.03 (dd, *J* = 2.0 Hz, 1.0 Hz, 1H, PyH_{2N}), 8.80 (d, *J* = 8.0 Hz, 1H, IleN*H*), 8.73 (d, *J* = 2.0 Hz, 1H, PyH_{2C}), 8.70 (dd, *J* = 5.0 Hz, 2.0 Hz, 1H, PyH_{6N}), 8.26 (dd, *J* = 5.0 Hz, 1.5 Hz, 1H, PyH_{6C}), 8.23 (ddd, *J* = 8.0 Hz, 2.0 Hz, 2.0 Hz, 1H, PyH_{4N}), 8.03 (ddd, *J* = 8.5 Hz, 2.5 Hz, 1.5 Hz, 1H, PyH_{4C}), 7.49 (ddd, *J* = 8.0 Hz, 5.0 Hz, 1.0 Hz, 1H, PyH_{5N}), 7.34 (dd, *J* = 8.5 Hz, 4.5 Hz, 1H, PyH_{5C}), 4.65 (dd, *J* = 8.5 Hz, 4.5 Hz, 1H, Pro(2)H_α), 4.53 (dd, *J* = 10 Hz, 8.0 Hz, 1H, IleH_α), 4.47 (dd, *J* = 8.0 Hz, 4.0 Hz, 1H, Pro(3)H_α), 3.99, 3.73, 3.61 (m, 4H, ProH_δ), 2.19, 2.06–1.82 (m, 9H, ProH_β, ProH_γ, IleH_β), 1.59 (ddd, *J* = 13 Hz, 8.0 Hz, 3.0 Hz, 1H, IleH_γ), 1.19 (m, 1H, IleH_γ), 0.96 (d, *J* = 7.0 Hz, 3H, Ile$H_{\gamma'}$), 0.84, (t, *J* = 7.5 Hz, 3H, IleH_δ), (*additional two sets of conformers exist*); **^{13}C-NMR** (125 MHz, DMSO-d_6, 300 K): δ 171.6 (Pro(3)*C*O), 170.4 (Pro(2)*C*O), 170.2 (Ile*C*O), 165.3 (Ile*C*O), 152.4 (PyC_{6N}), 149.2 (PyC_{2N}), 144.6 (PyC_{6C}), 141.0 (PyC_{2C}), 136.2 (PyC_{3C}), 135.8 (PyC_{4N}), 129.8 (PyC_{3N}), 126.3 (PyC_{4C}), 124.1 (PyC_{5C}), 123.8 (PyC_{5N}), 60.4 (Pro(3)C_α), 58.1 (Pro(2)C_α), 55.7 (IleC_α), 47.7, 47.2 (ProC_δ), 36.1 (IleC_β), 29.7, 28.5 (ProC_γ), 25.3 (IleC_γ), 25.1, 24.9 (ProC_β), 15.2 (Ile$C_{\gamma'}$), 10.9 (IleC_δ), (*additional two sets of conformers exist*).

Tripeptide ligand (**9**)

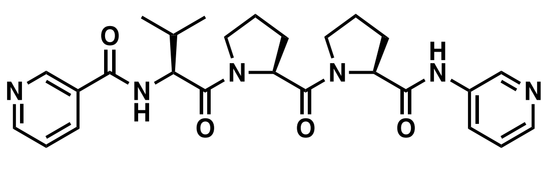

The product was obtained as a white solid 0.19 g (35% yield) following the same procedure for **3** and purified by column chromatography (EtOAc : MeOH = 10 : 1 → 2 : 1). The material was further purified by using a preparative SEC (W252 column, eluent: MeOH).

m.p.: 120.0–129.8 ºC; **ESI-MS** (*m/z*): $[M+Na]^+$ calcd for $C_{26}H_{32}N_6O_4Na$: 515.2, found: 515.3; **analysis**: (% calcd, % found for $C_{26}H_{32}N_6O_4{\bullet}(H_2O)_{1.5}$), C (60.10, 60.01), H (6.79, 6.68), N (16.17, 16.03); **^{1}H-NMR** (500 MHz, DMSO-d_6, 300 K): δ 10.26 (s, 1H, N*H*-Py), 9.03 (d, *J* = 1.5 Hz, 1H, PyH_{2N}), 8.76 (d, *J* = 8.0 Hz, 1H, ValN*H*), 8.73 (d, *J* = 2.0 Hz, 1H, PyH_{2C}), 8.71 (dd, *J* = 4.5 Hz, 1.5 Hz, 1H, PyH_{6N}), 8.26 (dd, *J* = 5.0 Hz, 1.5 Hz, 1H, PyH_{6C}), 8.23 (ddd, *J* = 8.0 Hz, 2.0 Hz, 2.0 Hz, 1H, PyH_{4N}), 8.03 (ddd, *J* = 8.0 Hz, 2.5 Hz, 1.5 Hz, 1H,

PyH_{4C}), 7.50 (dd, J = 7.0 Hz, 5.0 Hz, 1H, PyH_{5N}), 7.33 (d, J = 8.0 Hz, 5.0 Hz, 1H, PyH_{5C}), 4.65 (dd, J = 8.5 Hz, 5.0 Hz, 1H, Pro(2)H_{α}), 4.46 (m, 2H, ValH_{α}, Pro(3)H_{α}), 3.97, 3.73, 3.64–3.58 (m, 4H, ProH_{δ}), 2.2–1.8 (m, 8H, ProH_{β}, ProH_{γ}), 2.12 (m, 1H, ValH_{β}), 1.00, 0.97 (m, 6H, ValH_{γ}), (*additional two sets of conformers exist*); **^{13}C-NMR** (125 MHz, DMSO-d_6, 300 K): δ 171.6 (Pro(3)CO), 170.4 (CO), 170.1 (CO), 165.4 (Py-CO), 152.4 (PyC_{6N}), 149.2 (PyC_{2N}), 144.6 (PyC_{6C}), 141.0 (PyC_{2C}), 136.2 (PyC_{3C}), 135.8 (PyC_{4N}), 129.9 (PyC_{3N}), 126.3 (PyC_{4C}), 124.1 (PyC_{5C}), 123.8 (PyC_{5N}), 60.4 (Pro(3)C_{α}), 58.1 (Pro(2)C_{α}), 57.4 (ValC_{α}), 47.7, 47.2 (ProC_{δ}), 30.4 (ValC_{β}), 29.7, 28.5 (ProC_{γ}), 25.1, 24.9 (ProC_{β}), 19.7, 19.3 (ValC_{γ}), (*additional two sets of conformers exist*).

Supplementary Table

Supplementary Table 1 | Crystal data and structural refinement for **4**, **6**, and **8**

	**4**	**6**	**8**
Identification code	Saito88	Saito82	Saito60
CCDC number	1869043	1869042	1869041
Empirical formula	$C_{319.03}H_{401.81}Ag_{12}F_{24.09}N_{83}O$	$C_{288}H_{346}Ag_{12}F_{51}N_{72}O_{61}P_{8.50}$	$C_{330}H_{408}Ag_{12}B_{9}F_{36}N_{78}O_{92}$
Formula weight	9269.09	8319.05	9015.05
Temperature	108(2) K	103(2) K	100(2) K
Wavelength	0.71073 Å	0.71073 Å	0.71073 Å
Crystal system	Orthorhombic	Trigonal	Cubic
Space group	$P2_12_12$	$R3$	$F23$
Unit cell dimensions	a = 32.3232(19) Å	a = 23.268(2) Å	a = 33.843(3) Å
	b = 34.694(2) Å	b = 23.268(2) Å	b = 33.843(3) Å
	c = 23.2972(14) Å	c = 23.268(2) Å	c = 33.843(3) Å
	$\alpha = \beta = \gamma = 90°$	$\alpha = \beta = \gamma = 100.333(2)°$	$\alpha = \beta = \gamma = 90°$
Volume	26126(3) Å^3	11897 (3) Å^3	38762(5) Å^3
Z	2	1	4
Density (calculated)	1.178 Mg•m^{-3}	1.161 Mg•m^{-3}	1.545 Mg•m^{-3}
Absorption coefficient	0.551 mm^{-1}	0.590 mm^{-1}	0.698 mm^{-1}
F(000)	9473	4217	18460
Crystal size	0.45 × 0.25 × 0.11 mm^3	0.20 × 0.12 × 0.11 mm^3	0.05 × 0.05 × 0.03 mm^3
Theta range for data	1.59 to 25.79°	1.82 to 26.57°	1.70 to 19.79°
Index ranges	$-39 \leqq h \leqq 39$	$-28 \leqq h \leqq 28$	$-32 \leqq h \leqq 32$
Reflections collected	345520	153255	59670
Independent reflections	50066 [R_{int} = 0.0682]	32596 [R_{int} = 0.2364]	2958 [R_{int} = 0.0957]
Completeness	99.7% (θ = 19.79°)	98.9% (θ = 19.79°)	99.9% (θ = 19.79°)
Max. and min. transmission	0.9419 and 0.7896	0.9380 and 0.8912	0.9787 and 0.9666
Refinement method		Full-matrix least-squares on F^2	
Data / restraints / parameters	50066 / 628 / 2778	32596 / 618 / 1403	2958 / 20 / 197
Goodness-of-fit on F^2	1.072	0.919	1.340
Final R indices [$I > 2\sigma(I)$]	R_1 = 0.1072, wR_2 = 0.2804	R_1 = 0.1107, wR_2 = 0.2896	R_1 = 0.1029, wR_2 = 0.2816
R indices (all data)	R_1 = 0.14892, wR_2 = 0.3387	R_1 = 0.2619, wR_2 = 0.3574	R_1 = 0.1333, wR_2 = 0.3215
Absolute structure parameter	0.014(6)	0.090(14)	0.023(18)
Largest diff. peak and hole	1.982 and −1.251 e.Å^{-3}	1.149 and −0.580 e.Å^{-3}	1.387 and −0.647 e.Å^{-3}

Supplementary Figures

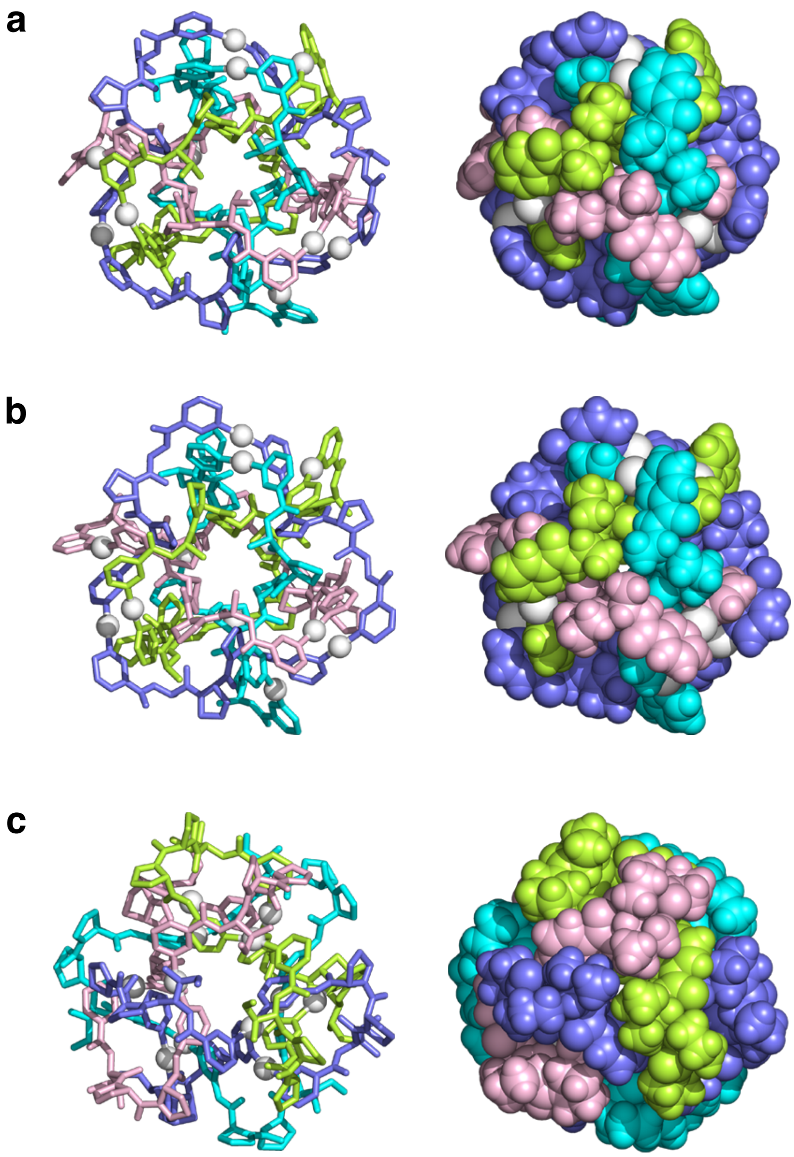

Supplementary Figure 1 | X-ray structures of $[4]_{12}$-catenanes. Stick (left) and space-filling (right) representations of $[4]_{12}$-catenanes. **a**, Three-crossed-type $[4]_{12}$-catenane **4**. **b**, Three-crossed-type $[4]_{12}$-catenane **6** obtained from APP-sequenced ligand **5** and $AgPF_6$. **c**, T_2-type $[4]_{12}$-catenane **8** obtained from IPP-sequenced ligand **7** and $AgBF_4$. Counter anions and solvents are omitted for clarity. Ag(I) ions are represented as white spheres.

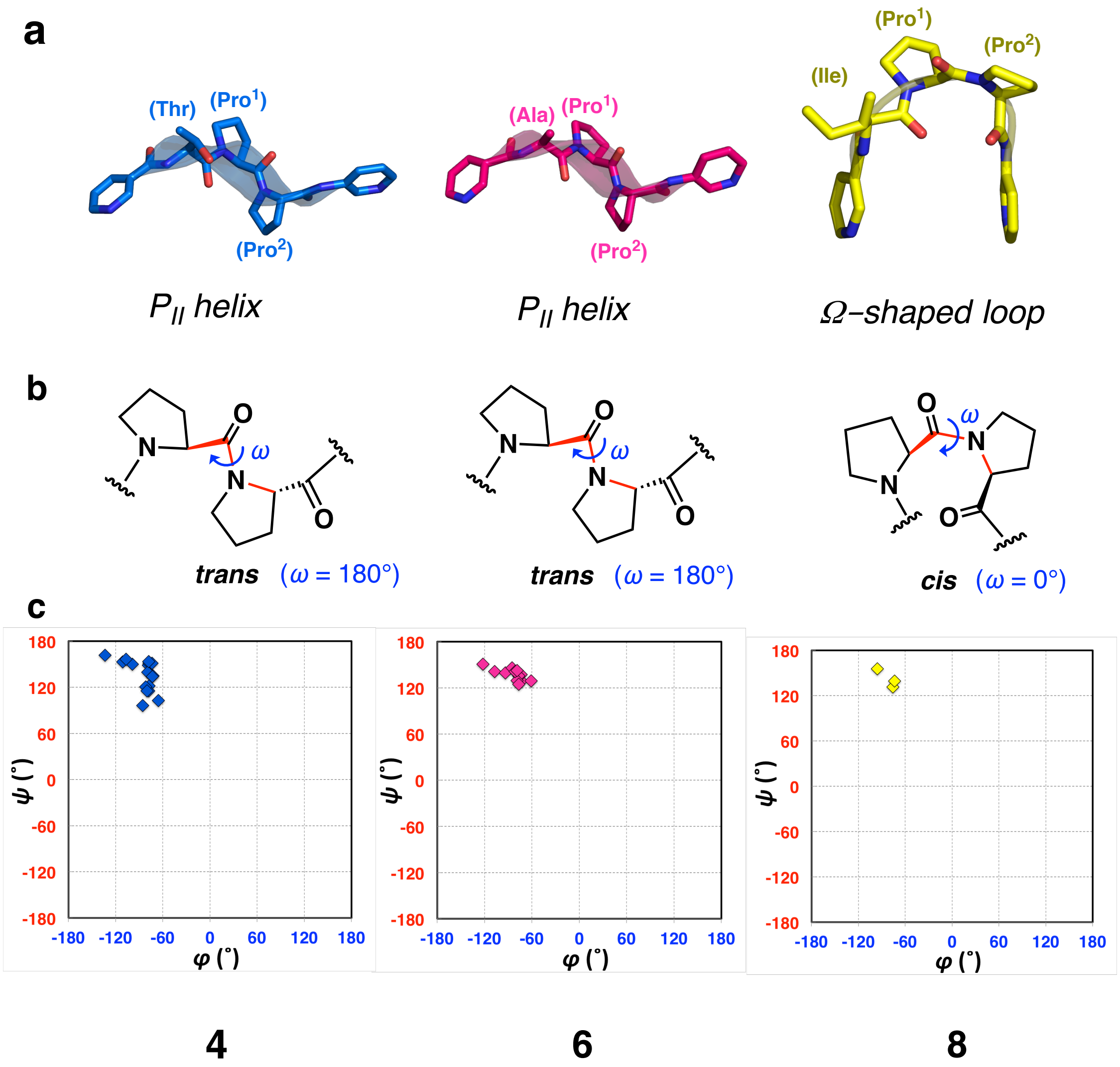

Supplementary Figure 2 | Peptide conformations. **a**, Typical peptide conformations observed in the crystal structures **4**, **6**, and **8**. **b**, The amide bond configurations between Pro-Pro residues. **c**, Ramachandran plots. Crystallographically independent six ligands for **3**, four ligands for **5** and one ligand for **7** were plotted respectively.

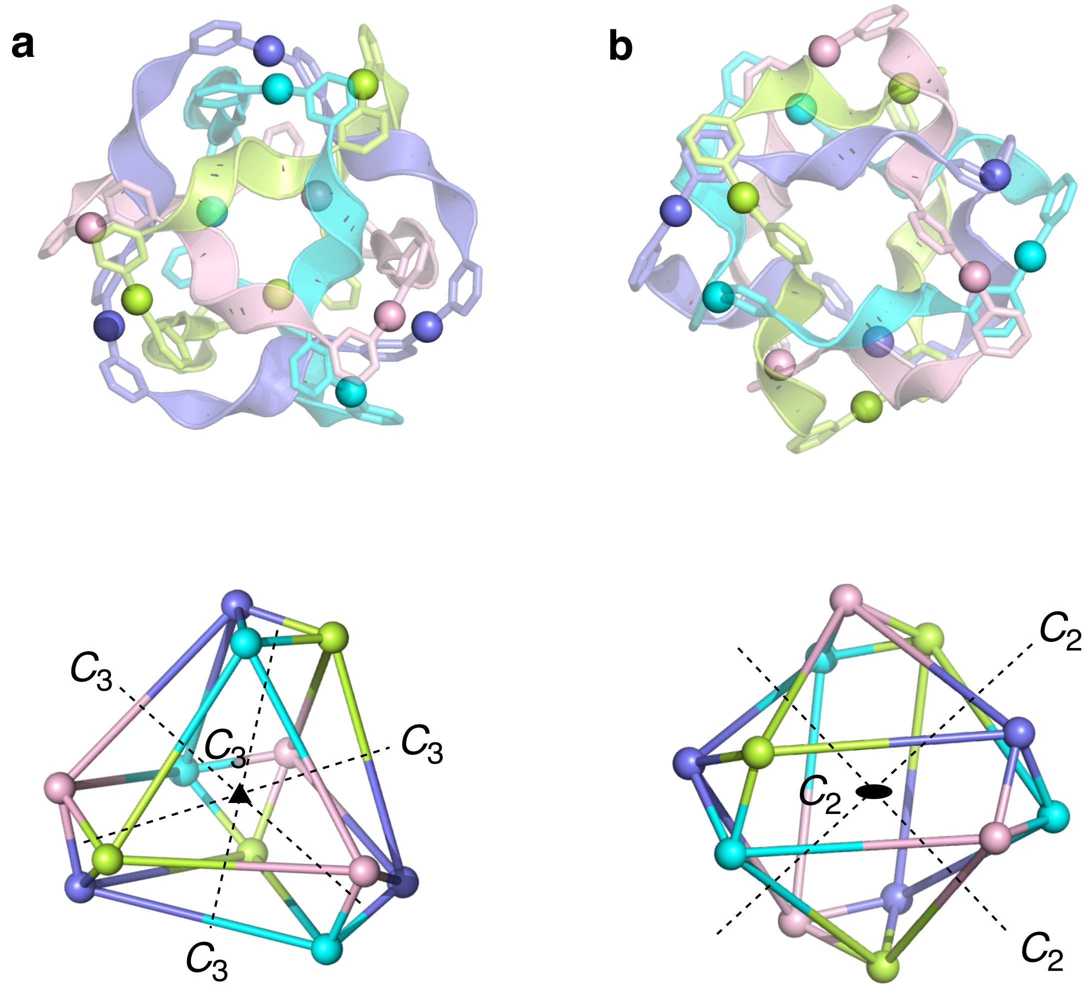

Supplementary Figure 3 | *T*-symmetry of 4. Four C_3 axis (**a**) and three C_2 axis (**b**) of the crystal structure of **4** are shown here. Cuboctahedron-like geometry of Ag(I) positions were extracted to below.

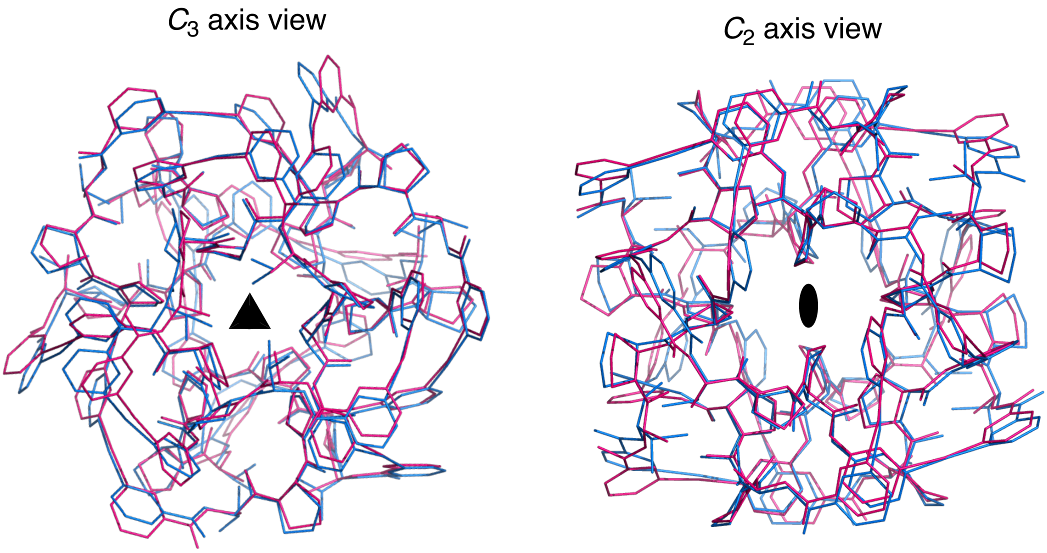

Supplementary Figure 4 | Overlaid structures of three-crossed [4]$_{12}$-catenanes. Two crystal structures, **4** (*blue*) and **6** (*red*) are overlaid in line representation.

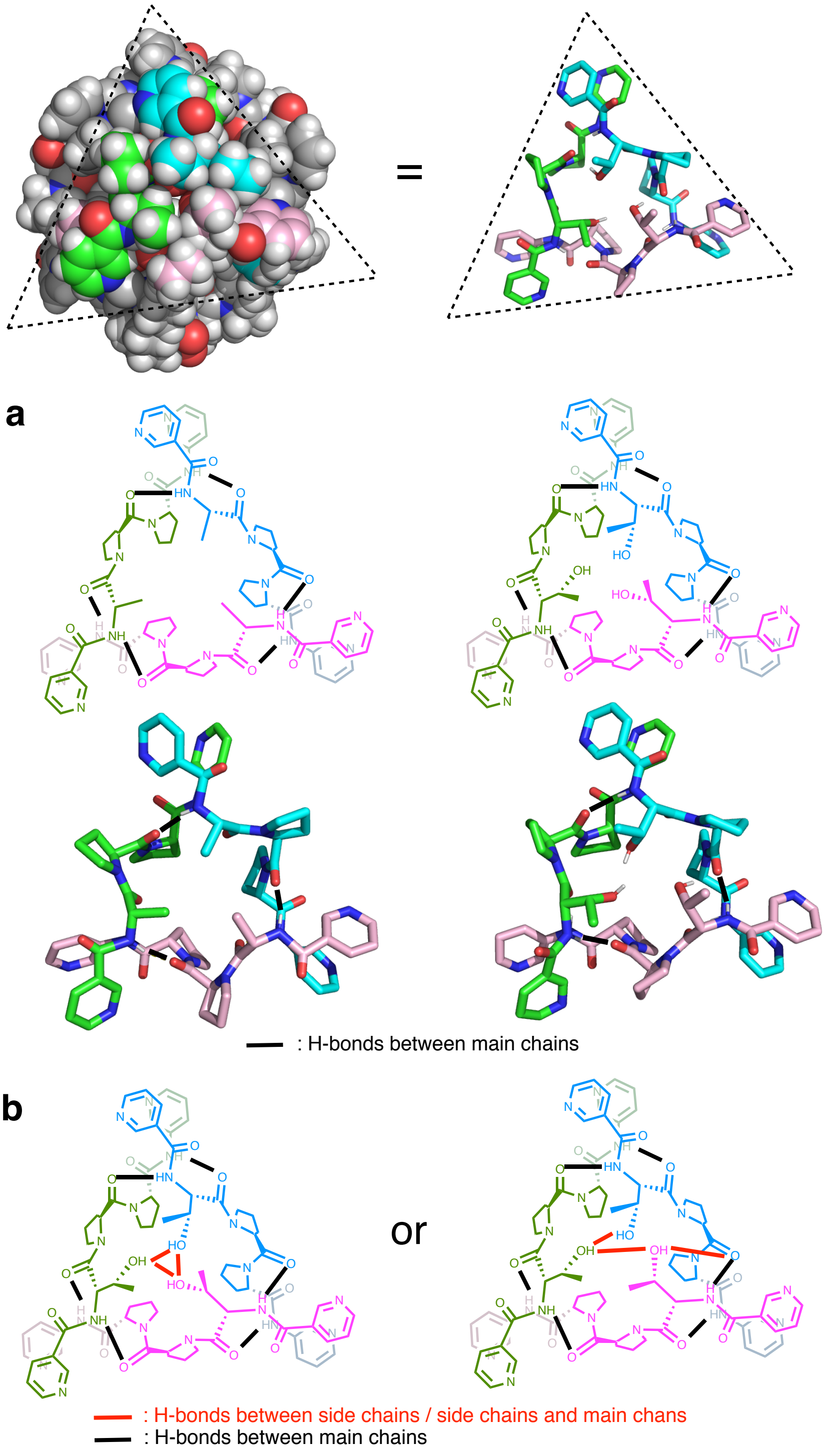

Supplementary Figure 5 | H-bonds in the three-crossed geometry. a, Six sets of H-bonds between mains chains are observed in the structure of **6** and **4**. **b**, Six sets of H-bonds between main chains and three sets between side chains (threonine) are observed in the structure of **4**. H-bonds between side chains and main chains are also observed in the structure due to the flexibility of the ligands.

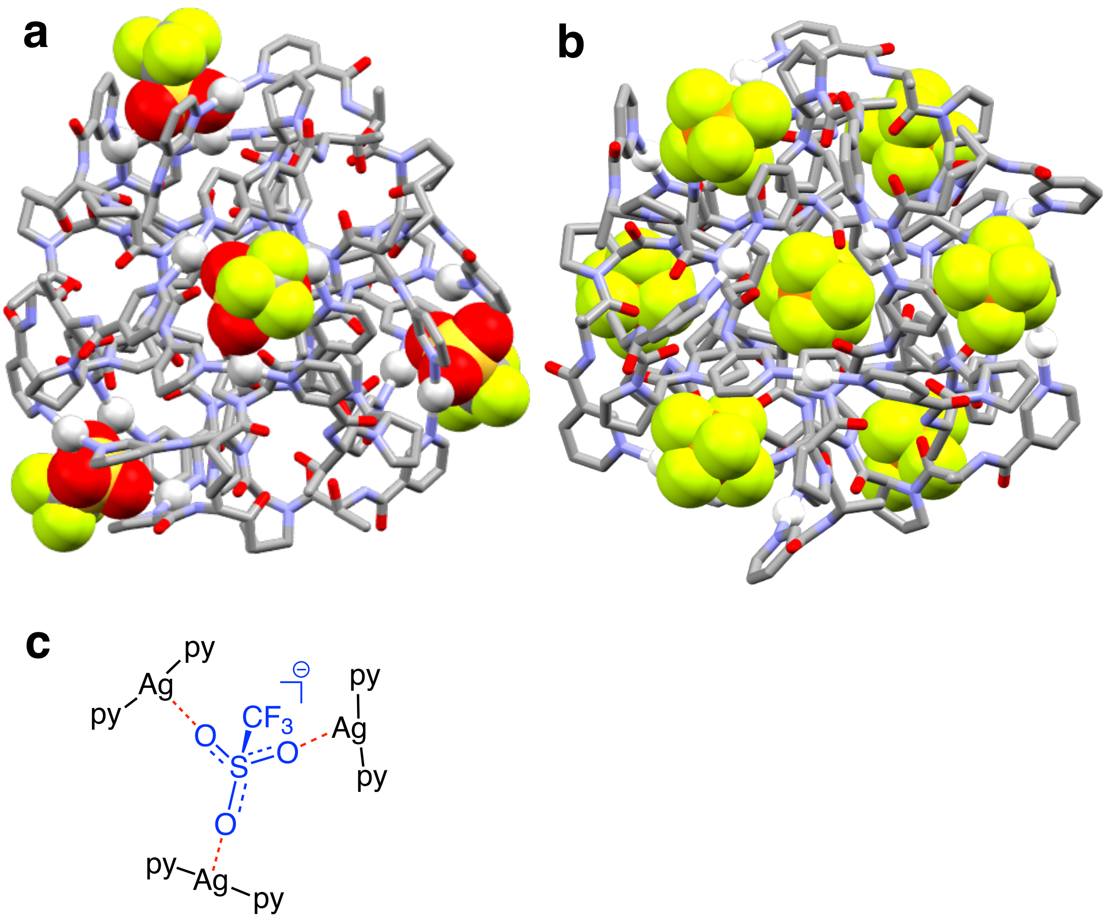

Supplementary Figure 6 | Anions positions in the framework of the three-crossed $[4]_{12}$-catenanes. a, Triflate (OTf^-) anions in the structure of **4** are highlighted in space-filing representation. **b**, PF_6^- anions in the structure of **6** are highlighted in the space-filing representation. **c**, C_3 symmetry of triflate anion templates the triangle structure of Ag(I) in **4**, which suggests the possible reason for the quantitative $[4]_{12}$-catenane formation only in the case of triflate anion (see also Supplementary Fig. 15).

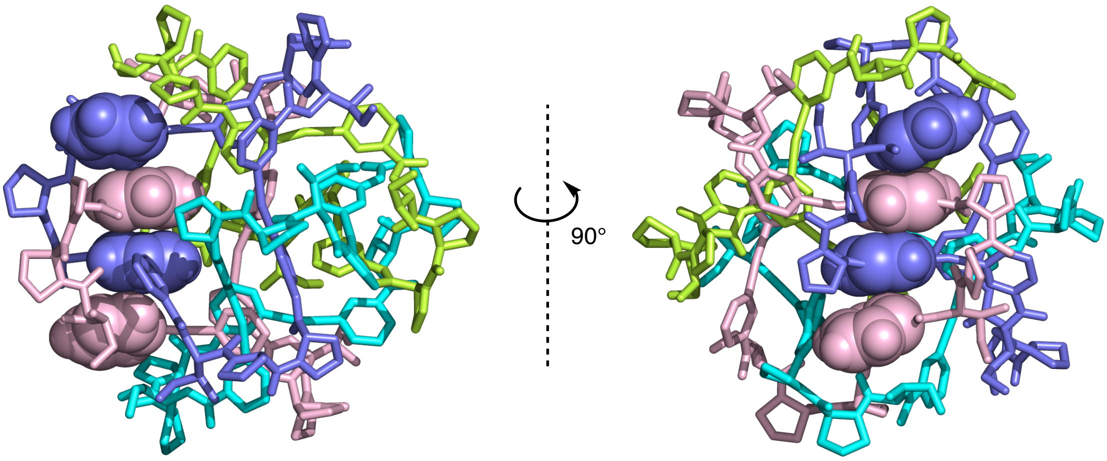

Supplementary Figure 7 | Quadruple Stacking of pyridyl groups in the structure of *T_2*-[4]$_{12}$-catenane 8**.** Pyridyl groups highlighted in space-filing representation indicate quadruple stacking inducing the T_2-edge geometry similar to **2**.[2]

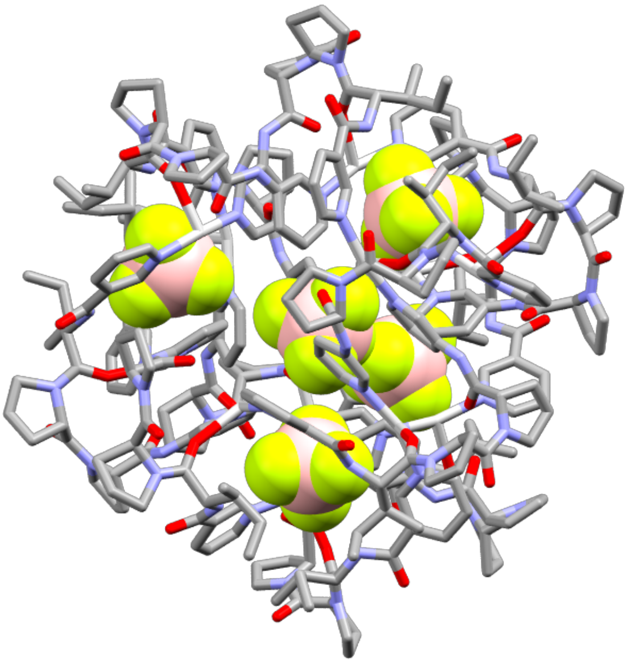

Supplementary Figure 8 | BF_4^- positions in the framework of *T_2*-[4]$_{12}$-catenane 8**.** Five BF_4^-anions highlighted in space-filling representation induced the T_2-tetrahedral link framework. H-bonds are observed between anions and amide on the framework of the [4]$_{12}$-catenane as seen in the structure of **2**.[2]

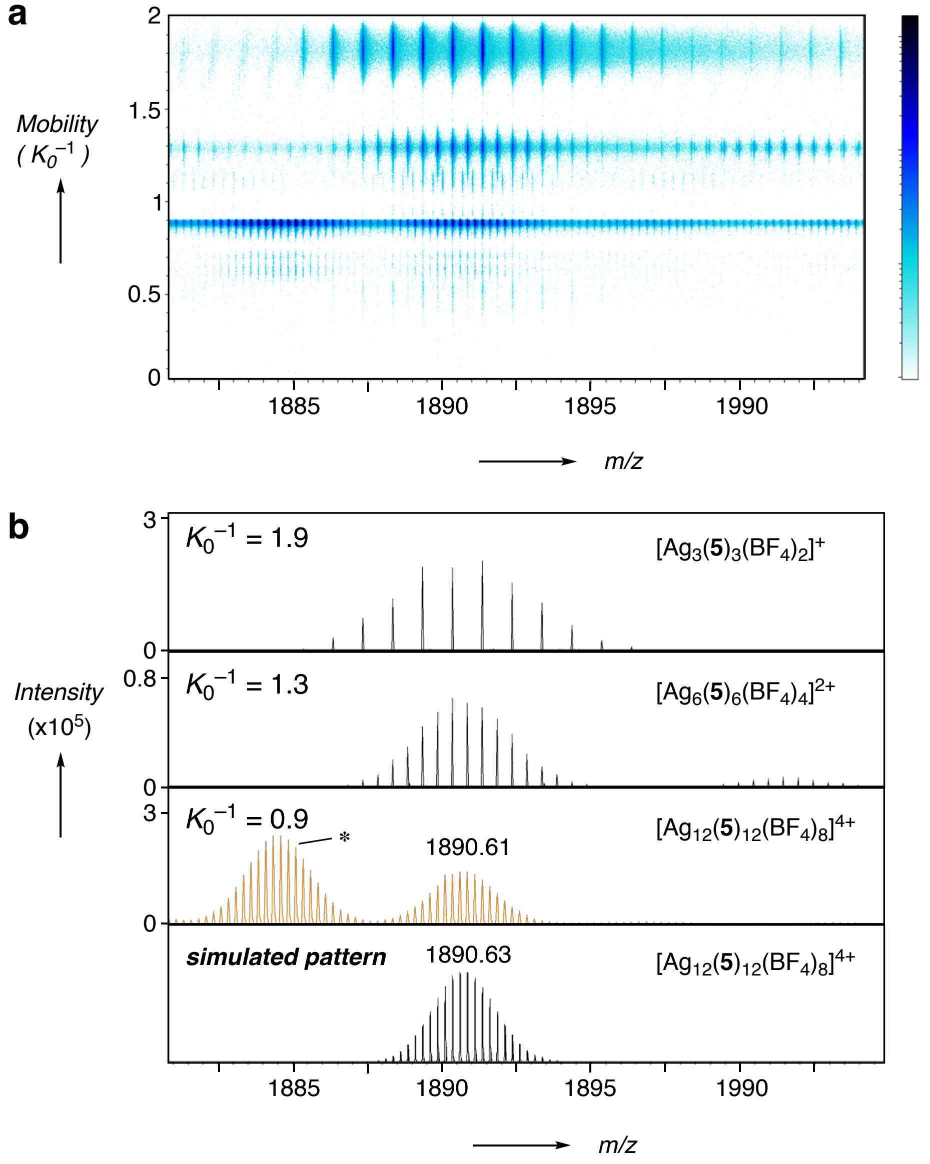

Supplementary Figure 9 | Ion mobility mass spectrometry data of [4]$_{12}$-catenane 6. **a**, Mobility vs. *m/z* plot. **b**, Mass spectra at K_0^{-1} = 1.9, K_0^{-1} = 1.3, K_0^{-1} = 0.9, and simulated pattern of $[Ag_{12}(\mathbf{5})_{12}(BF_4)_8]^{4+}$. The set of peaks marked with an asterisk corresponds to $[Ag_{12}(\mathbf{5})_{12}(BF_4)_7(NO_3)]^{4+}$, which is derived from a small quantity of $AgNO_3$ in the reagent grade $AgBF_4$.

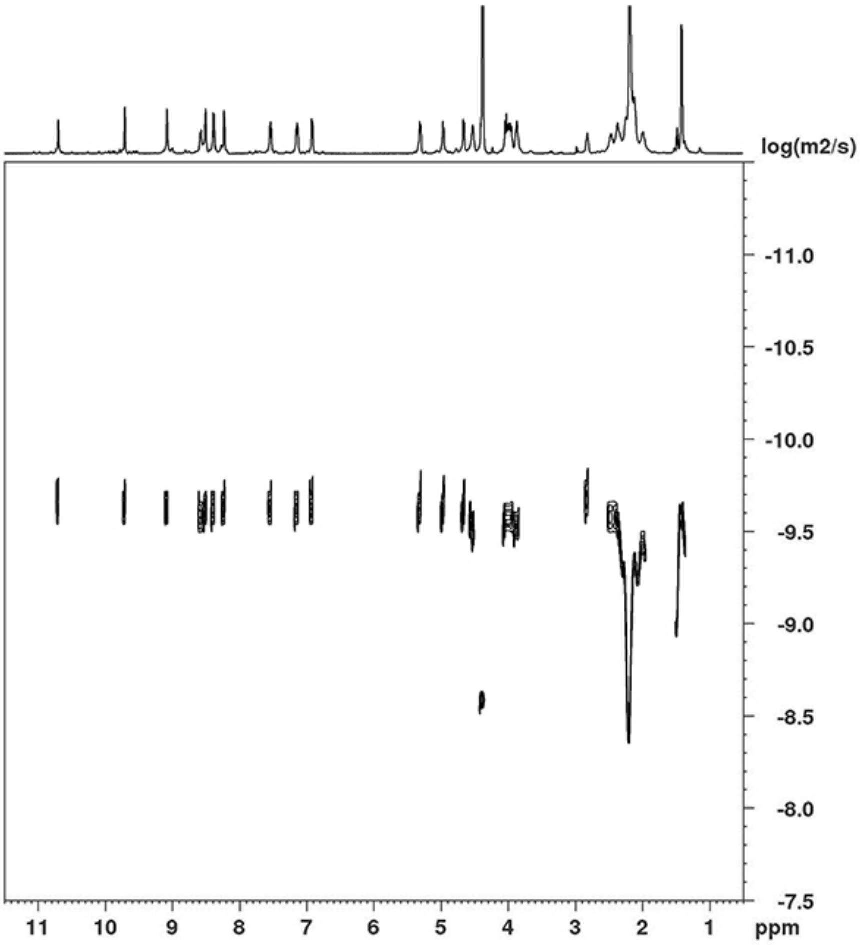

Supplementary Figure 10 | ^{1}H DOSY spectrum of [4]$_{12}$-catenane 4. The spectrum indicates a single product with a diffusion coefficient D of 2.4×10^{-10} m^2 s^{-1} (log$D$ = −9.62). 500 MHz, CD_3NO_2, 300 K.

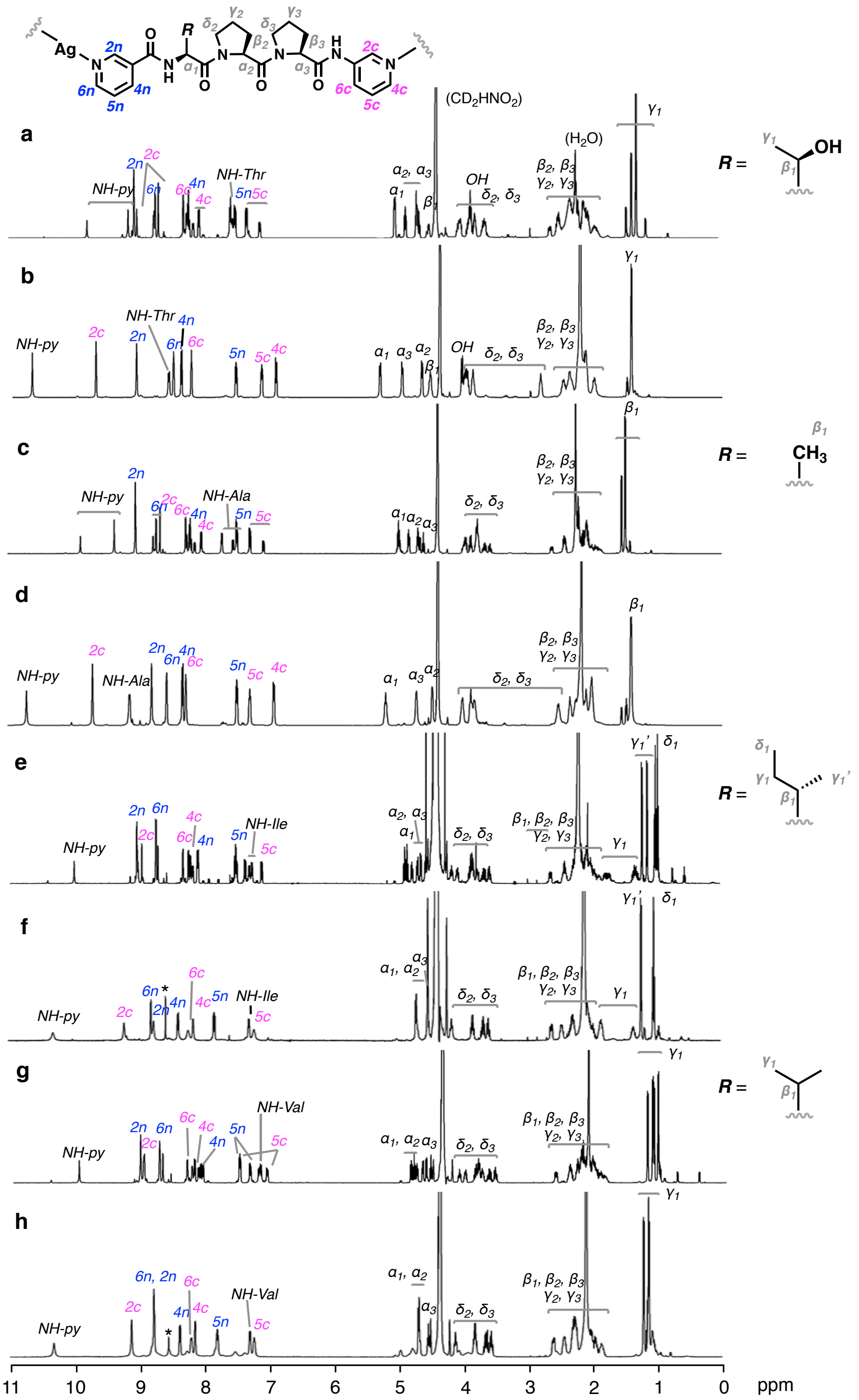

Supplementary Figure 11 | ^{1}H NMR spectra before and after self-assembly of [4]$_{12}$-catenanes. ^{1}H NMR spectra (500 MHz, CD_3NO_2, 300 K) of **a**, ligand **3**, **b**, [4]$_{12}$-catenane **4** (counter anion: OTf^-), **c**, ligand **5**, **d**, [4]$_{12}$-catenane **6** (counter anion: OTf_2^-), **e**, ligand **7**, **f**, [4]$_{12}$-catenane **8** (counter anion: BF_4^-), **g**, ligand **9**, and **h**, [4]$_{12}$-catenane **10** (counter anion: BF_4^-). Note that conformers exist in **a**, **c**, **e**, and **g** owing to the structural flexibility of the short peptide ligands. The signals derived from CD_3NO_2 are marked with asterisks.

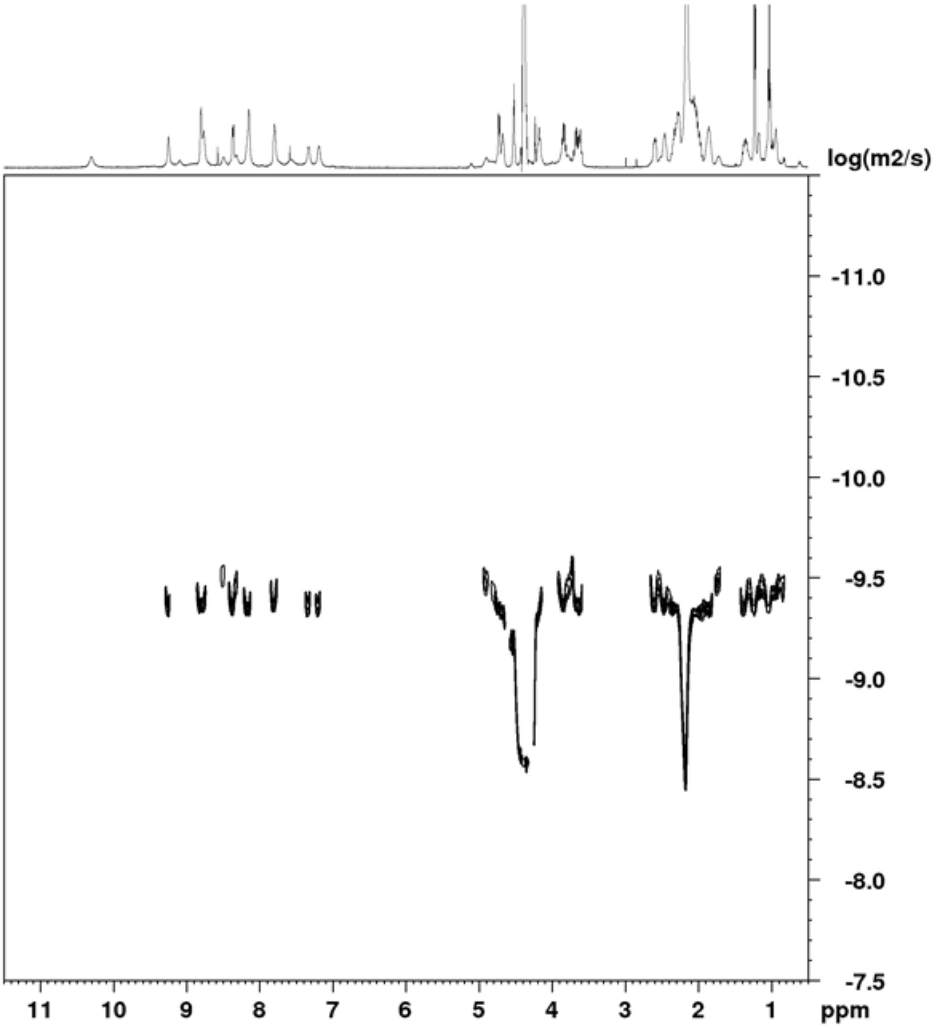

Supplementary Figure 12 | ^{1}H DOSY spectrum of [4]$_{12}$-catenane 8. The spectrum indicates a single product with a diffusion coefficient *D* of 4.3×10^{-10} m^2 s^{-1} (log*D* = −9.36). 500 MHz, CD_3NO_2, 300 K.

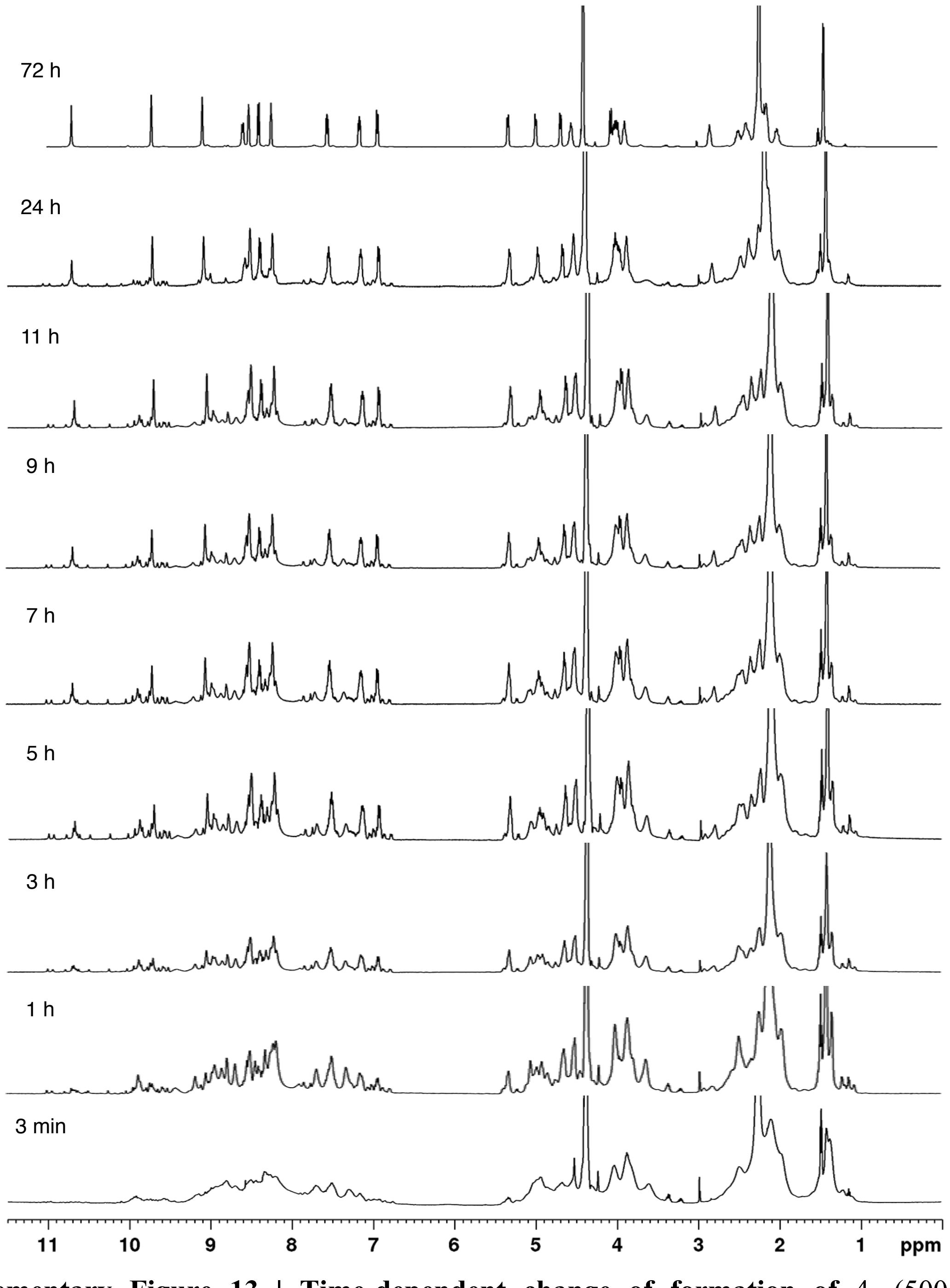

Supplementary Figure 13 | Time-dependent change of formation of 4. (500 MHz, CD_3NO_2). For initial 3 min ~ 11 h, reaction temperature was kept at 323 K in order to speed up the change. For 24 ~ 72 h, that was kept at 300 K. The broad signals just after mixing of ligand **3** and Ag(I) solution starts to change into 10 peaks at aromatic regions in 3 h, then completely converged in 72 h.

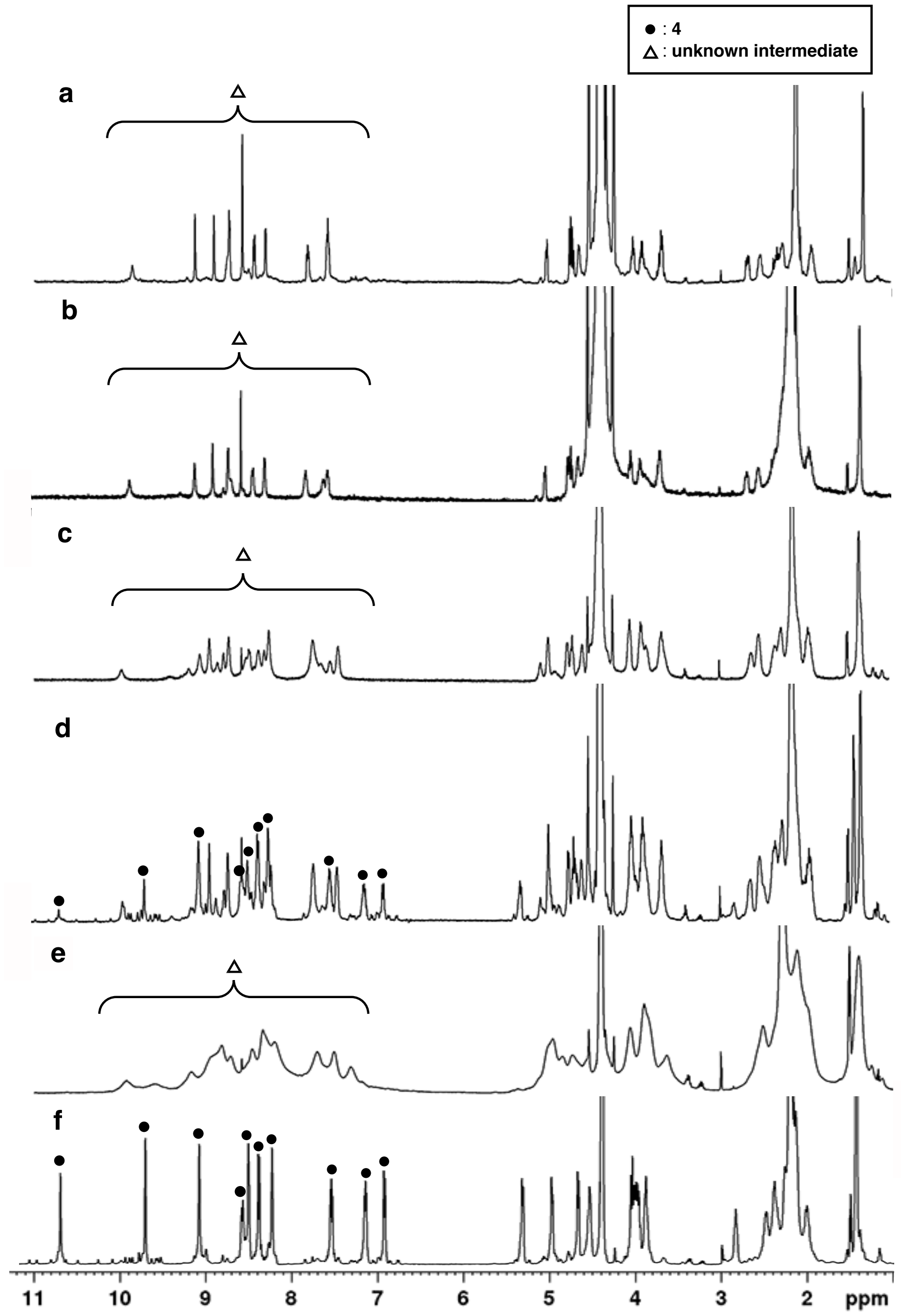

Supplementary Figure 14 | Initial concentration effect on formation of 4. (500 MHz, CD_3NO_2, 300 K). **a**, 1 mM (just after mixing). **b**, 1 mM (after standing for 2 d). **c**, 10 mM (just after mixing). **d**, 10 mM (after standing for 2 d). **e**, 50 mM (just after mixing). **f**, 50 mM (after standing for 2 d). Higher concentration (50 mM) is preferred to formation of **4**. At lower concentration (~10 mM), complexation is trapped into the stable intermediates (the structure is unknown).

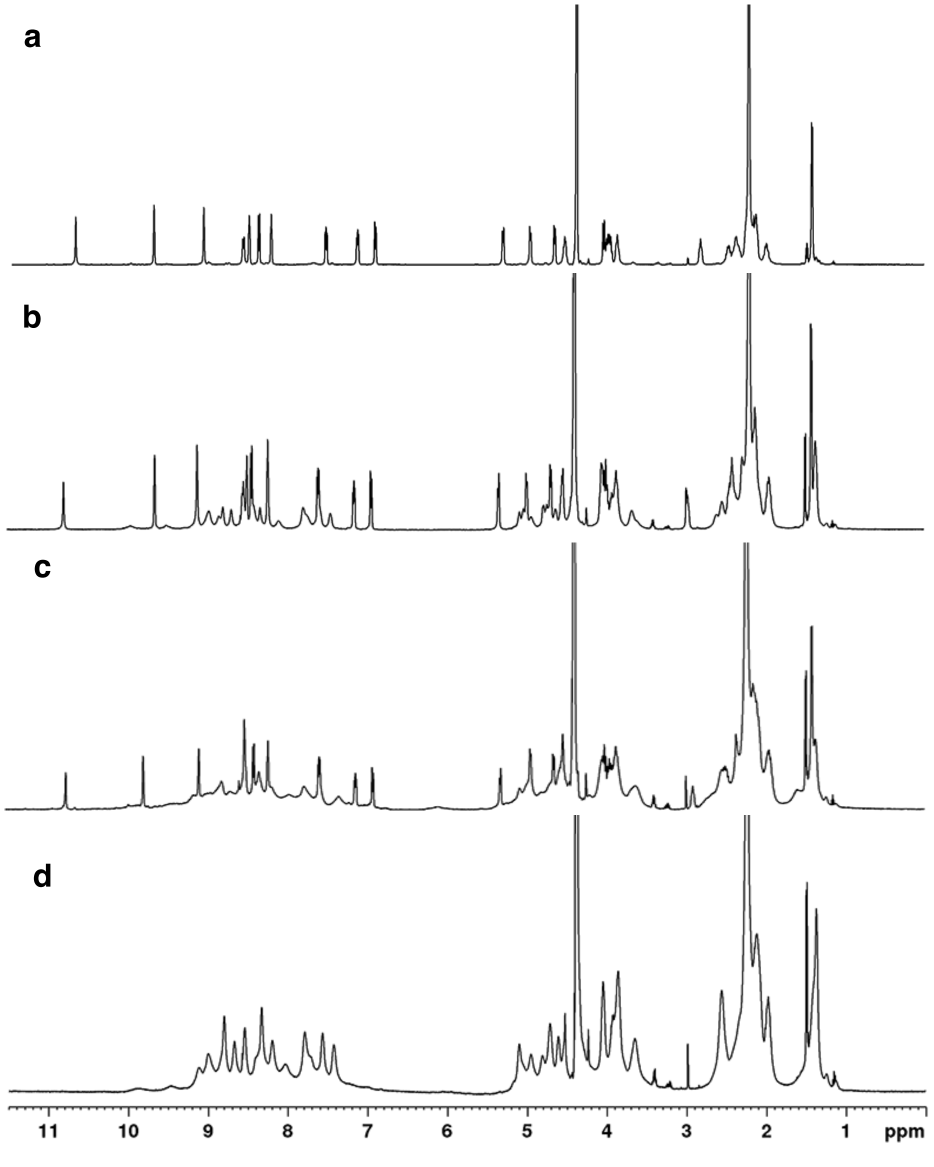

Supplementary Figure 15 | Counter anion dependence on formation of 4. (500 MHz, CD_3NO_2, 300 K). **a**, **3**+AgOTf. **b**, **3**+$AgPF_6$. **c**, **3**+$AgBF_4$. **d**, **3**+$AgNTf_2$. ([**3**] = [Ag^+] = 50 mM, after standing at r.t. for *ca* 2 d). The spectra **b** and **c** shows that minor broad peaks remained among the major sharp peaks. The spectrum **d** shows no converged pattern. These results revealed that OTf^- was the most suitable counter anion for inducing three-crossed $[4]_{12}$-catenane framework **4**. See also Supplementary Fig. 6c.

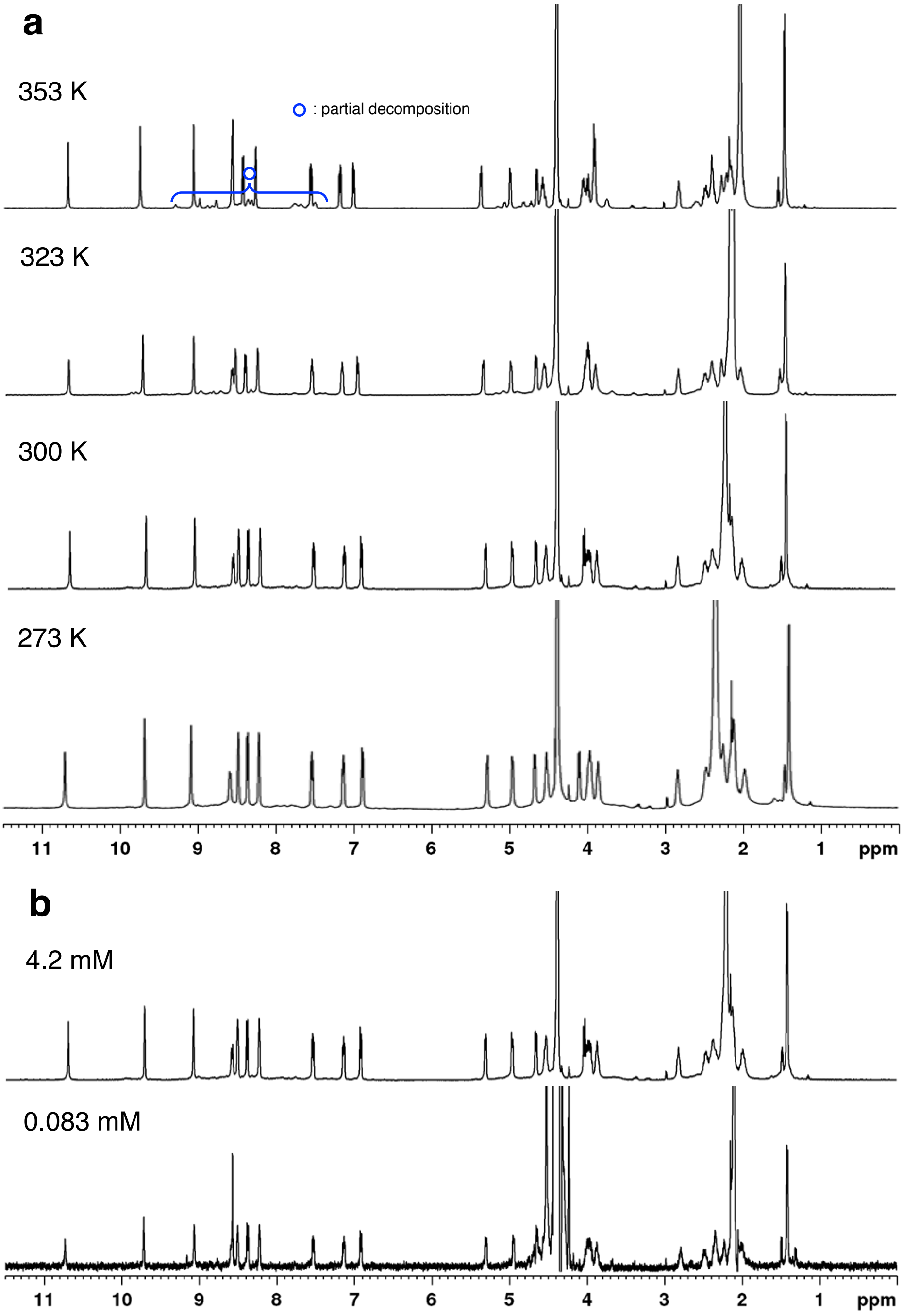

Supplementary Figure 16 | Stability test of 4 **in solution state.** (500 MHz, CD_3NO_2, 300 K). **a**, Temperature stability of **4**. The signal pattern does not show significant change from 273 K to 353 K without partial decomposition at 353 K. **b**, Dilution test after the formation of **4** ([**4**] = 4.2 mM to 8.3 $\times 10^{-2}$ mM).

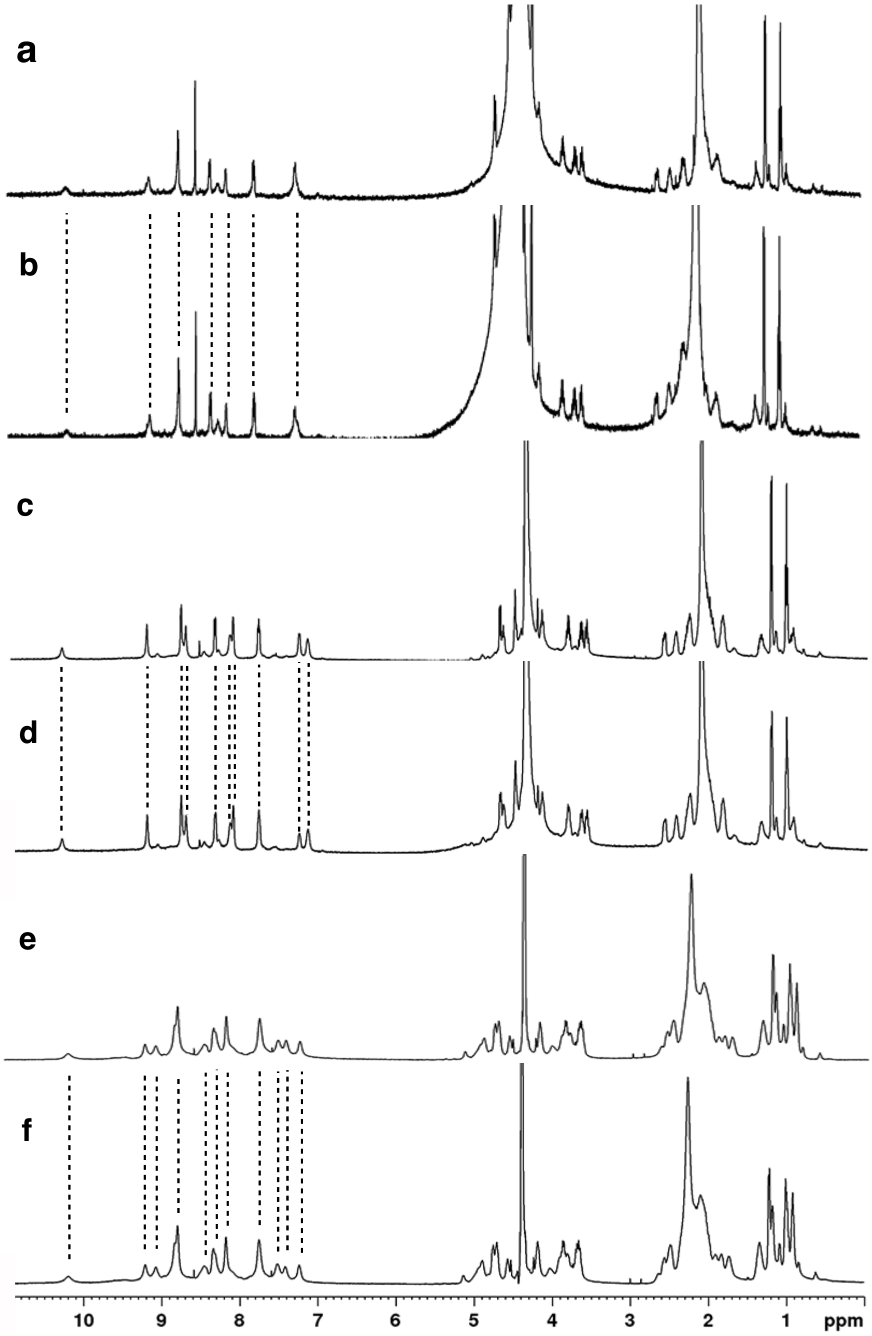

Supplementary Figure 17 | Initial concentration effect on formation of 8. (500 MHz, CD_3NO_2, 300 K). **a**, 1 mM (just after mixing), **b**, 1 mM (after standing for 2 d), **c**, 10 mM (just after mixing), **d**, 10 mM (after standing for 2 d), **e**, 50 mM (just after mixing), **f**, 50 mM (after standing for 2 d). Lower concentration (≦10 mM) is suitable for T_2-$[4]_{12}$-catenane formation. Compared to the case of three-crossed $[4]_{12}$-catenane, no significant initial concentration effect and time-dependent change are observed in this case.

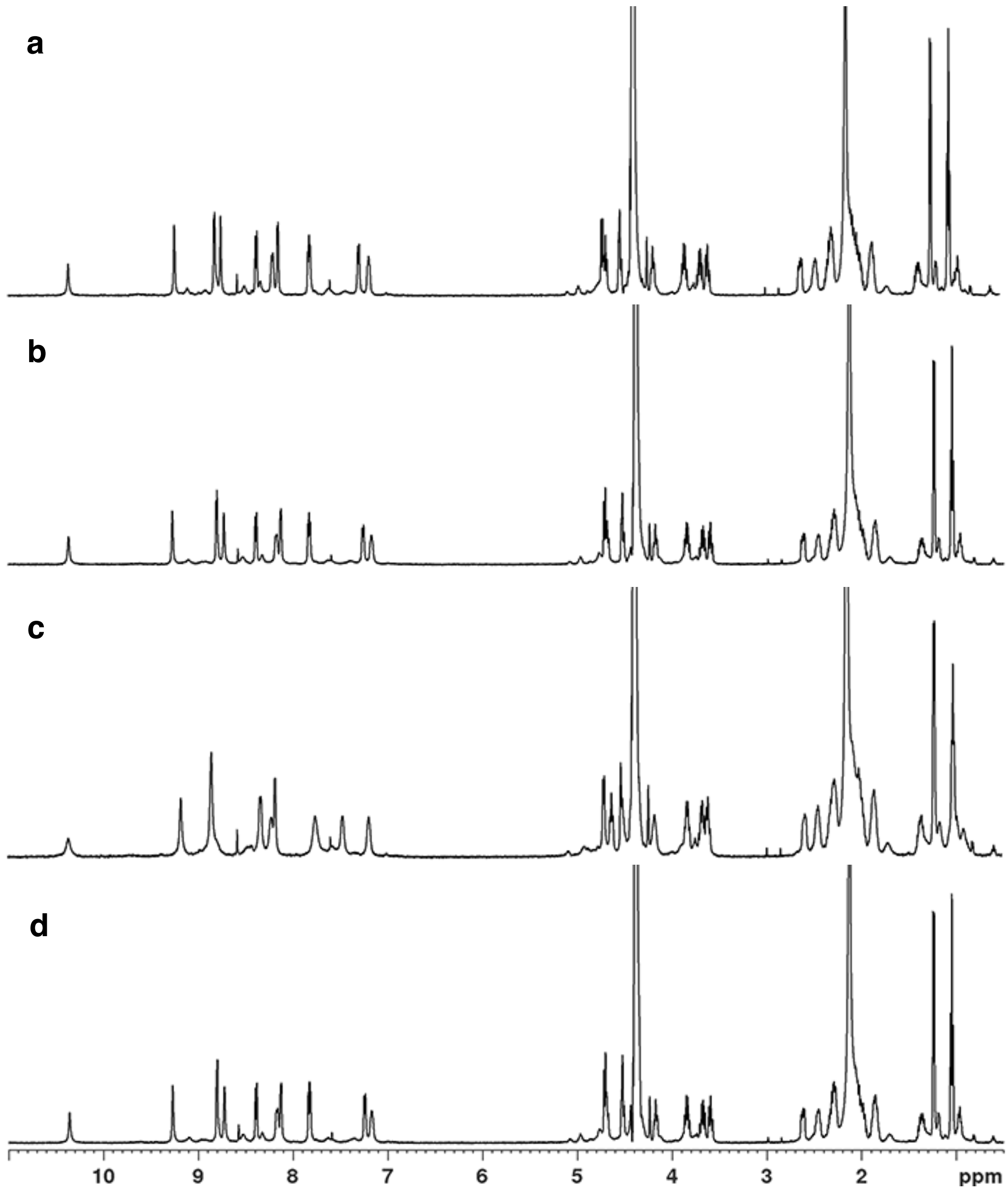

Supplementary Figure 18 | Counter anion dependence on formation of 8**.** (500 MHz, CD_3NO_2, 300 K). **a**, **7**+$AgBF_4$, **b**, **7**+$AgPF_6$, **c**, **7**+AgOTf, **d**, **7**+$AgNTf_2$, ([**7**] = [Ag^+] = 10 mM, mixed for 1 min by using a vortex mixer). Similar pattern among **a**, **b** and **c** suggests that the shape of counter anion is not dominant for the formation of **8**.

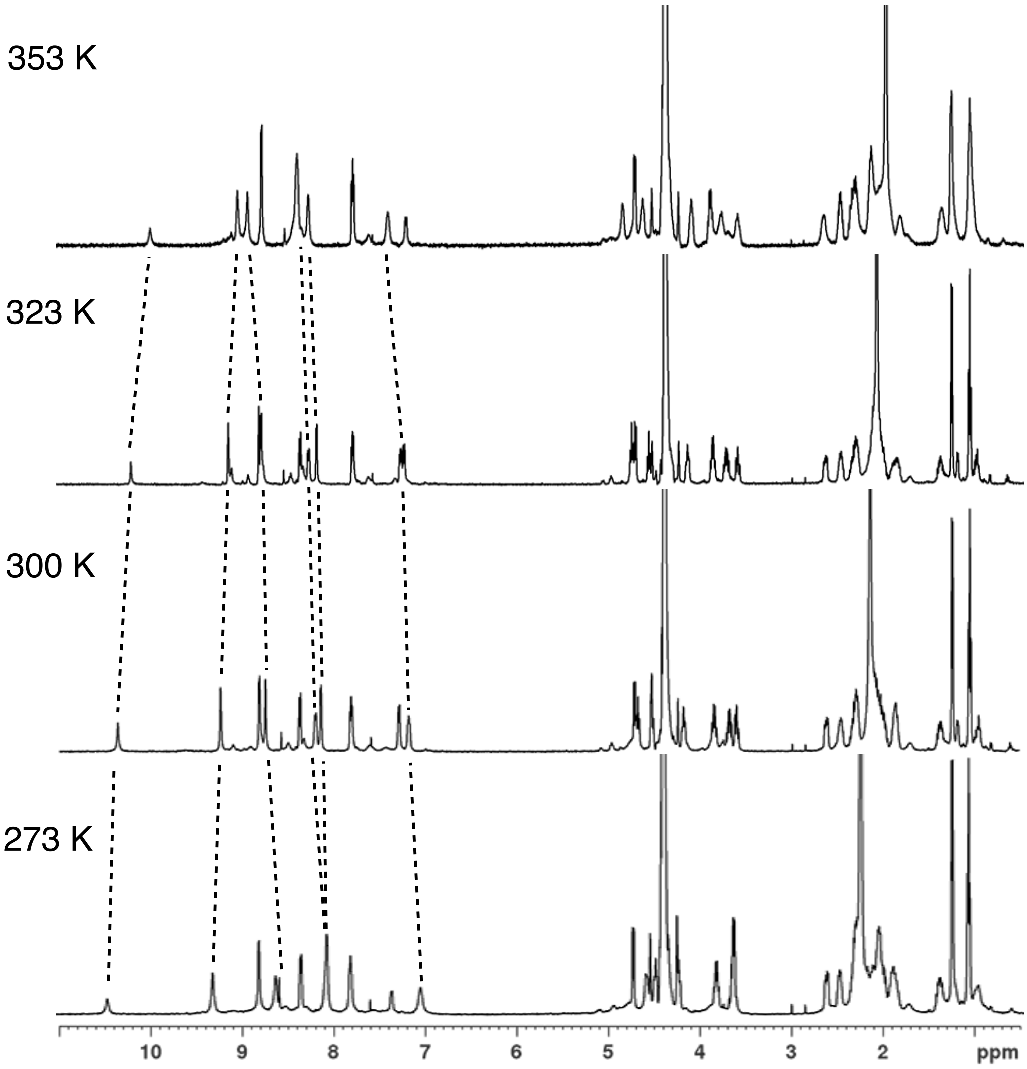

Supplementary Figure 19 | Temperature dependence on 8. (500 MHz, CD_3NO_2, [**7**] = $[Ag^+]$ = 10 mM, mixed for 1 min by using vortex). Dashed lines show the peak shifts. Unlike the case of three-crossed $[4]_{12}$-catenane (see also Supplementary Fig. 16a), slight peak shifts are observed depending on measurement temperature in the case of T_2-$[4]_{12}$-catenane.

Characterisation of **4**

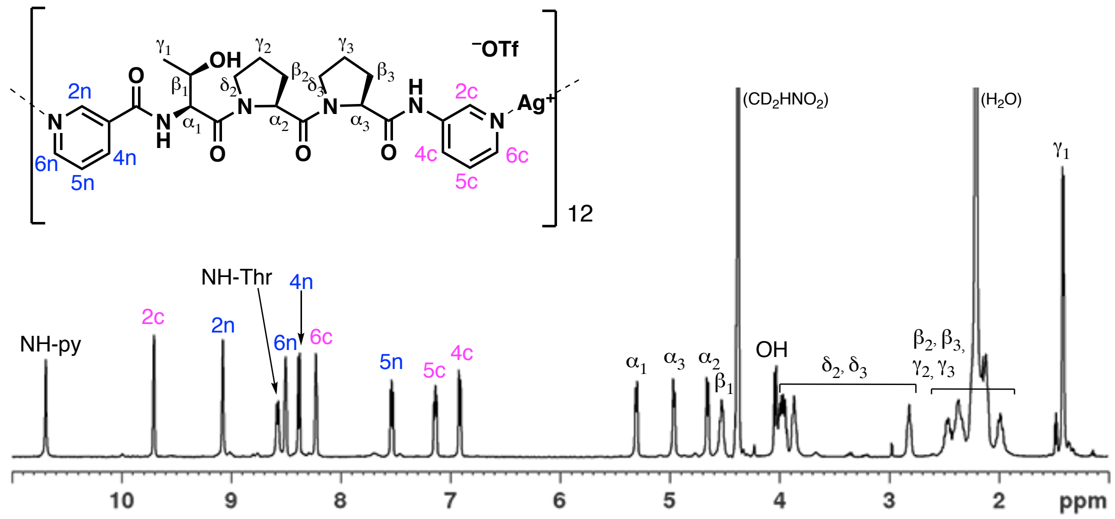

Supplementary Figure 20 | ^{1}H NMR spectrum of **4** (counter anion: OTf^-) (500 MHz, CD_3NO_2, 300 K).

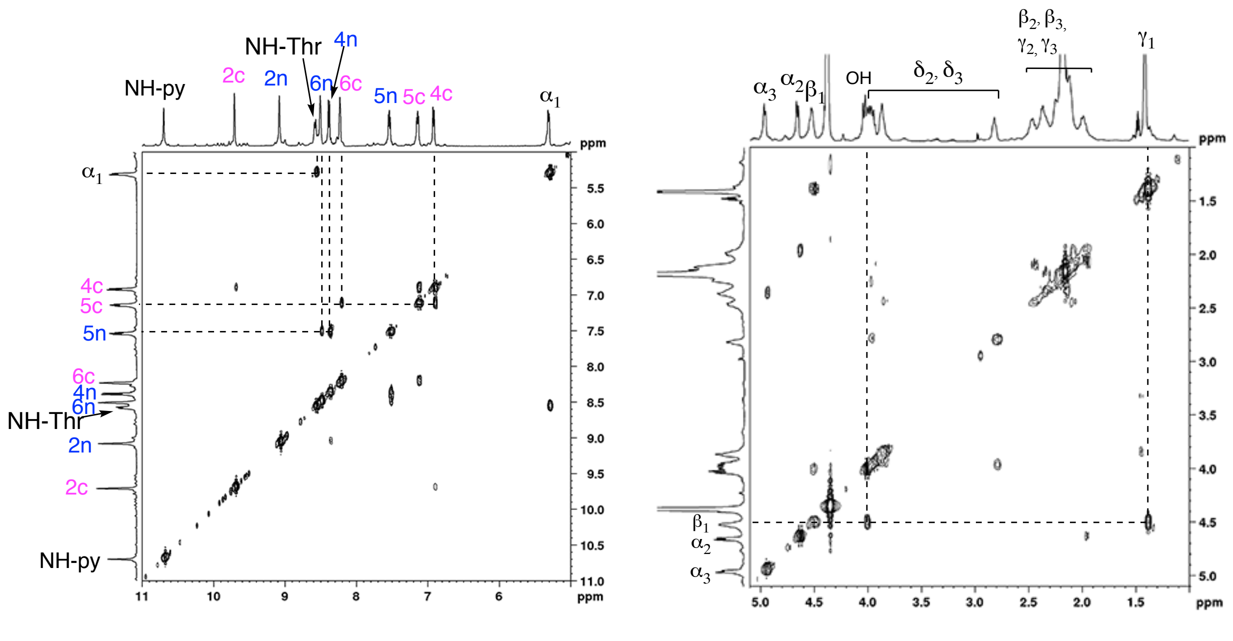

Supplementary Figure 21 | ^{1}H–^{1}H COSY spectra of **4** (counter anion: OTf^-) (500 MHz, CD_3NO_2, 300 K).

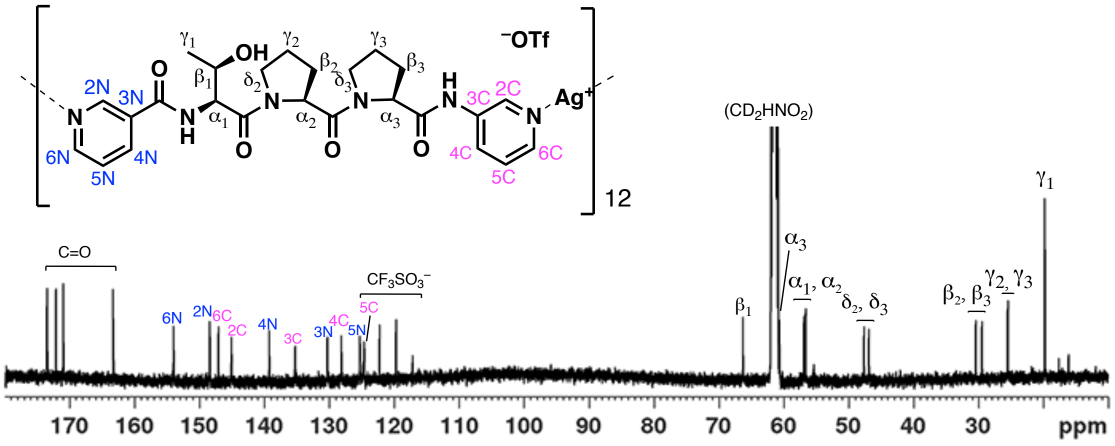

Supplementary Figure 22 | ^{13}C NMR spectrum of **4** (counter anion: OTf^-) (125 MHz, CD_3NO_2, 300 K).

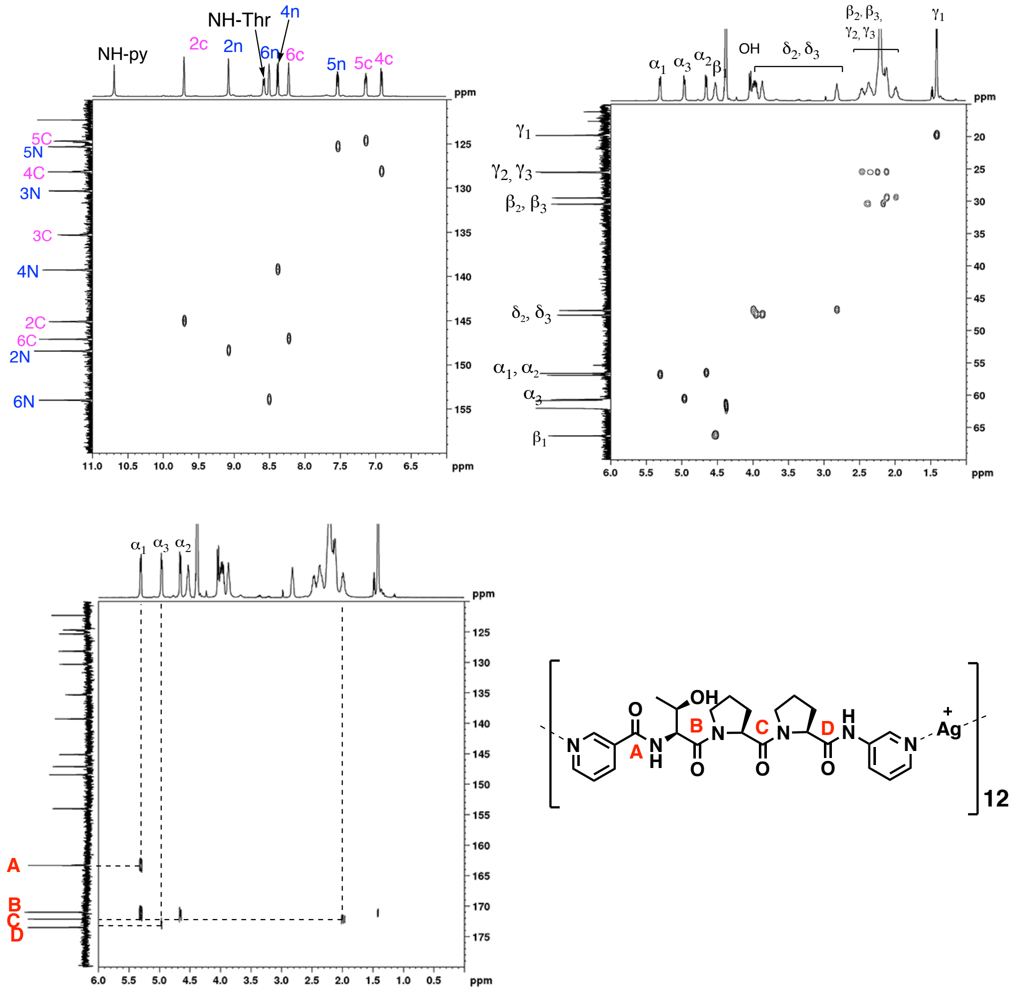

Supplementary Figure 23 | ^{1}H–^{13}C HSQC spectra (upper) and HMBC spectrum (bottom) of **4** (counter anion: OTf^-) (CD_3NO_2, 300 K).

Characterisation of **6**

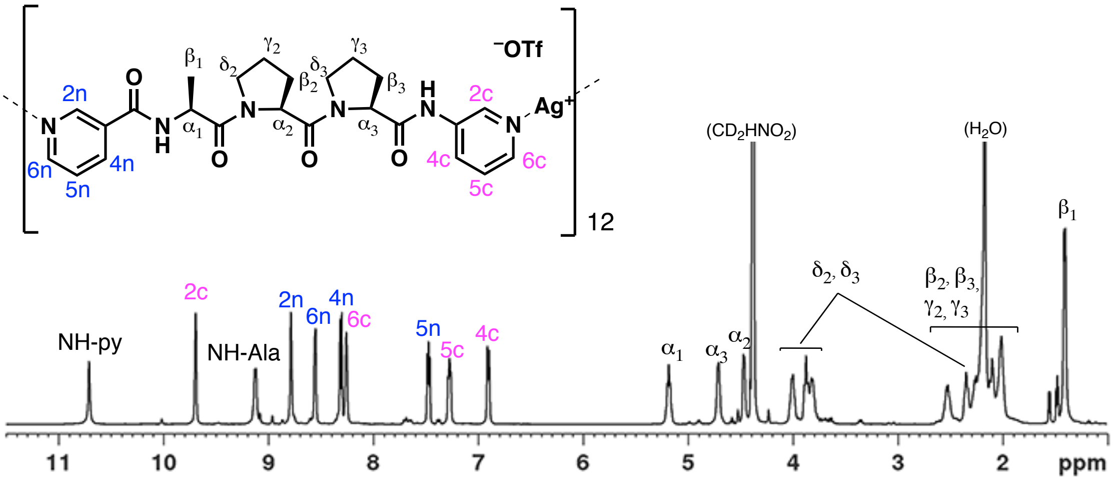

Supplementary Figure 24 | ^{1}H NMR spectrum of **6** (counter anion: OTf^{-}) (500 MHz, CD_3NO_2, 300 K).

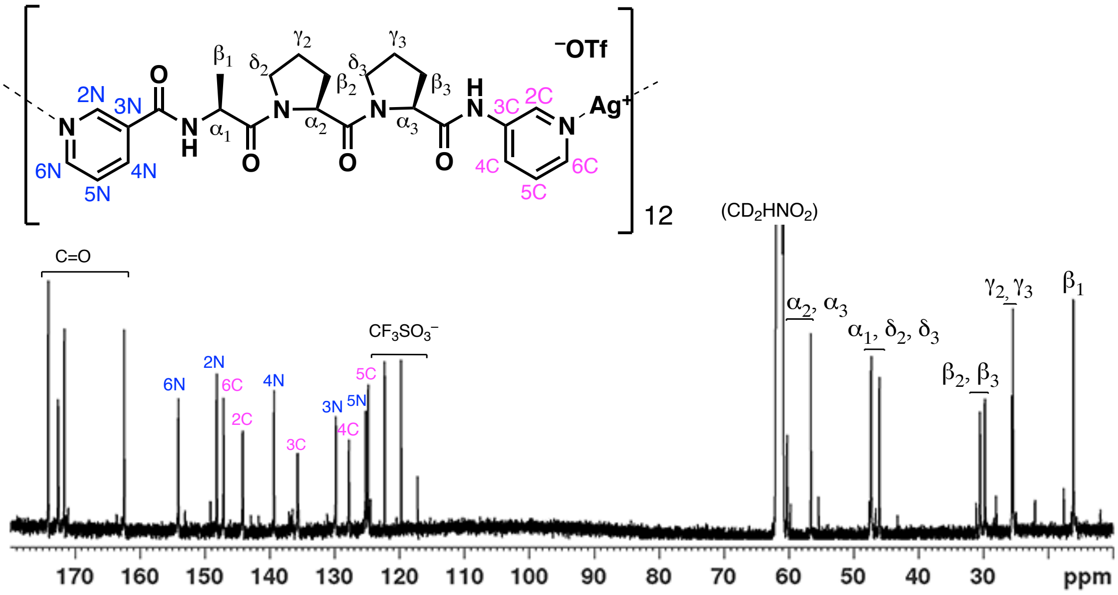

Supplementary Figure 25 | ^{13}C NMR spectrum of **6** (counter anion: OTf^{-}) (125 MHz, CD_3NO_2, 300 K).

Characterisation of **8**

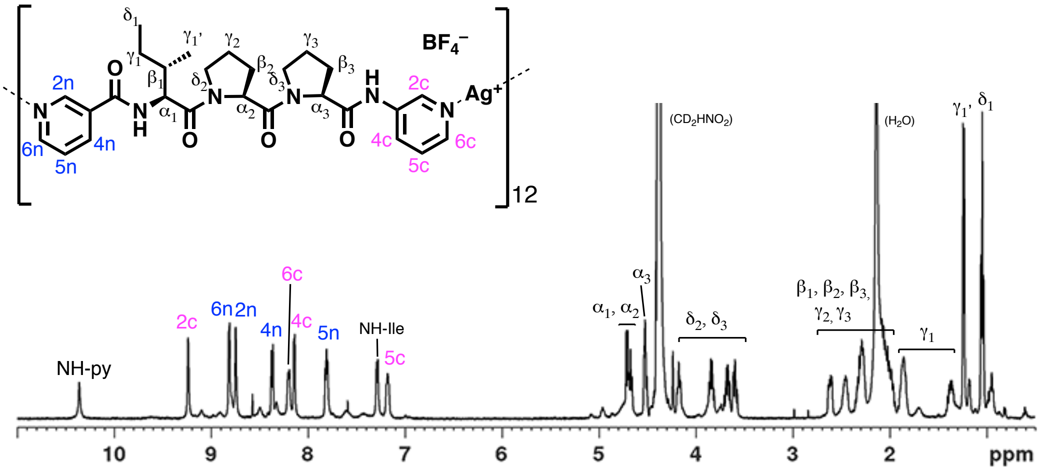

Supplementary Figure 26 | ^{1}H NMR spectrum of **8** (counter anion: BF_4^-) (500 MHz, CD_3NO_2, 300 K).

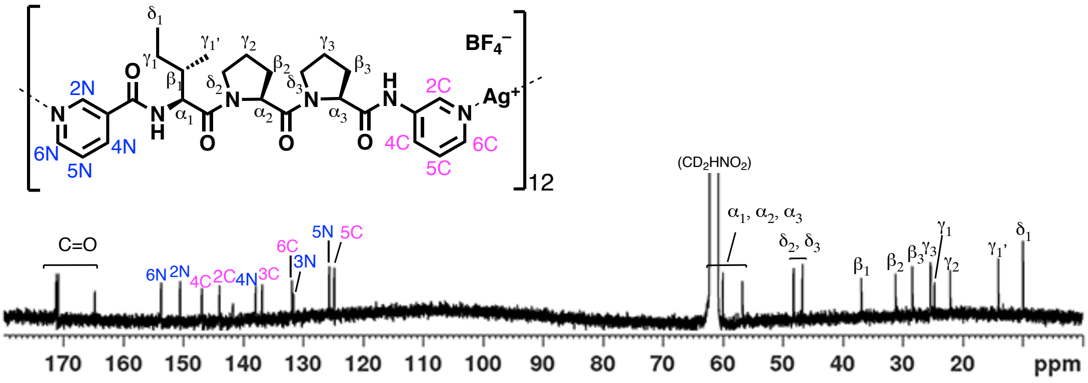

Supplementary Figure 27 | ^{13}C NMR spectrum of **8** (counter anion: BF_4^-) (125 MHz, CD_3NO_2, 300 K).

Characterisation of **10**

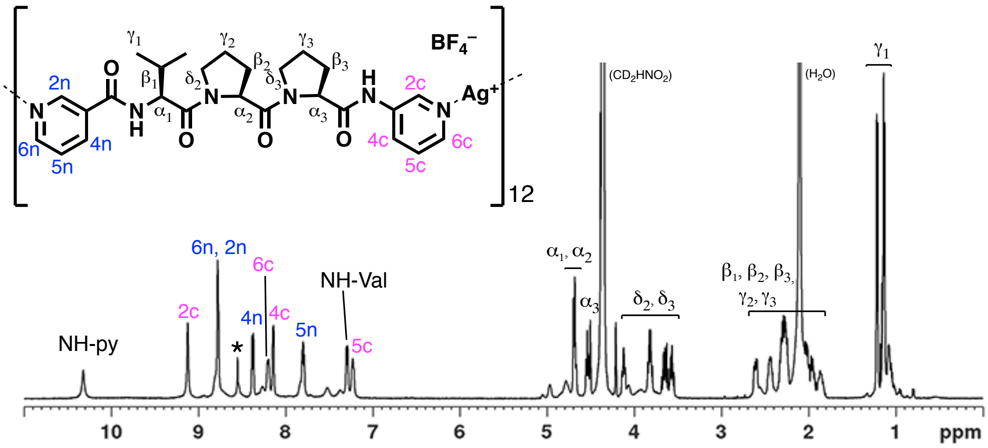

Supplementary Figure 28 | ^{1}H NMR spectrum of **10** (counter anion: BF_4^-) (500 MHz, CD_3NO_2, 300 K). The signal derived from CD_3NO_2 are indicated by asterisk.

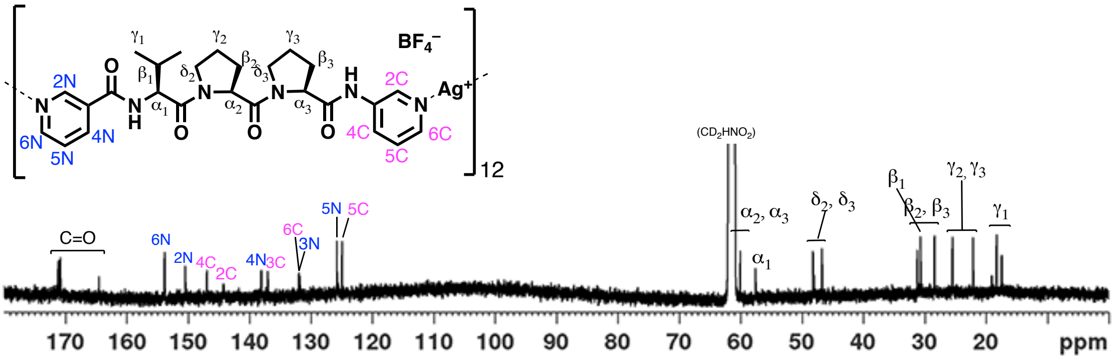

Supplementary Figure 29 | ^{13}C NMR spectrum of **10** (counter anion: BF_4^-) (125 MHz, CD_3NO_2, 300 K).

NMR Spectra of S2–S5 **and ligands** 3**,** 5**,** 7, and 9

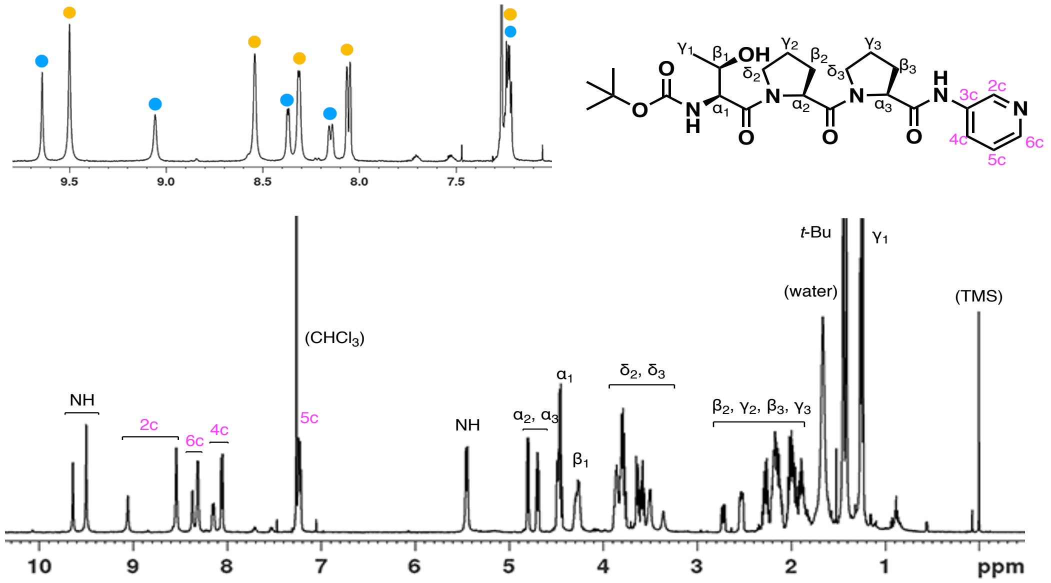

Supplementary Figure 30 | ^{1}H NMR spectrum (500 MHz, $CDCl_3$, 300 K) of **S2** (conformers exist).

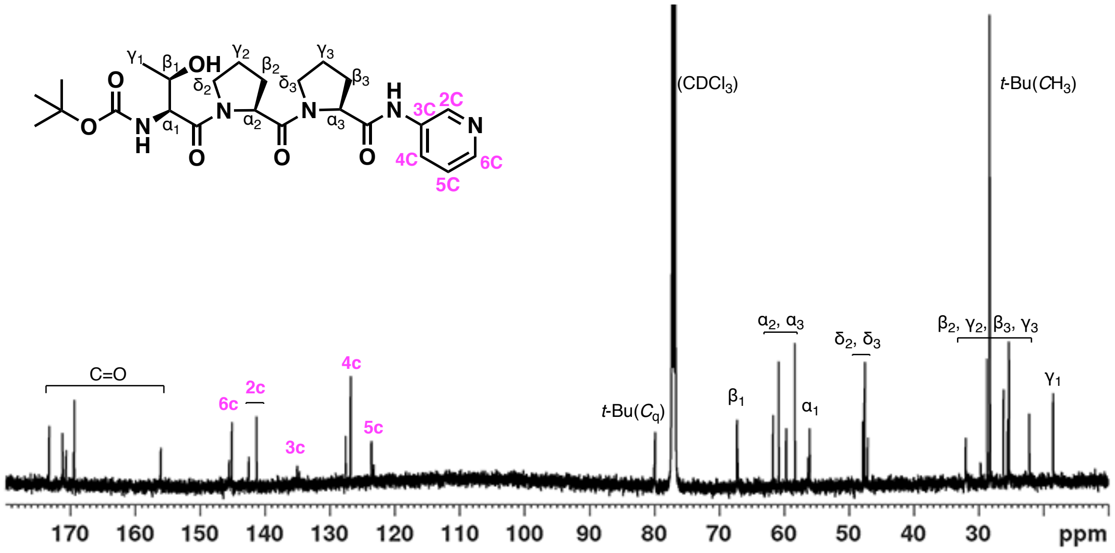

Supplementary Figure 31 | ^{13}C NMR spectrum (125 MHz, $CDCl_3$, 300 K) of **S2** (conformers exist).

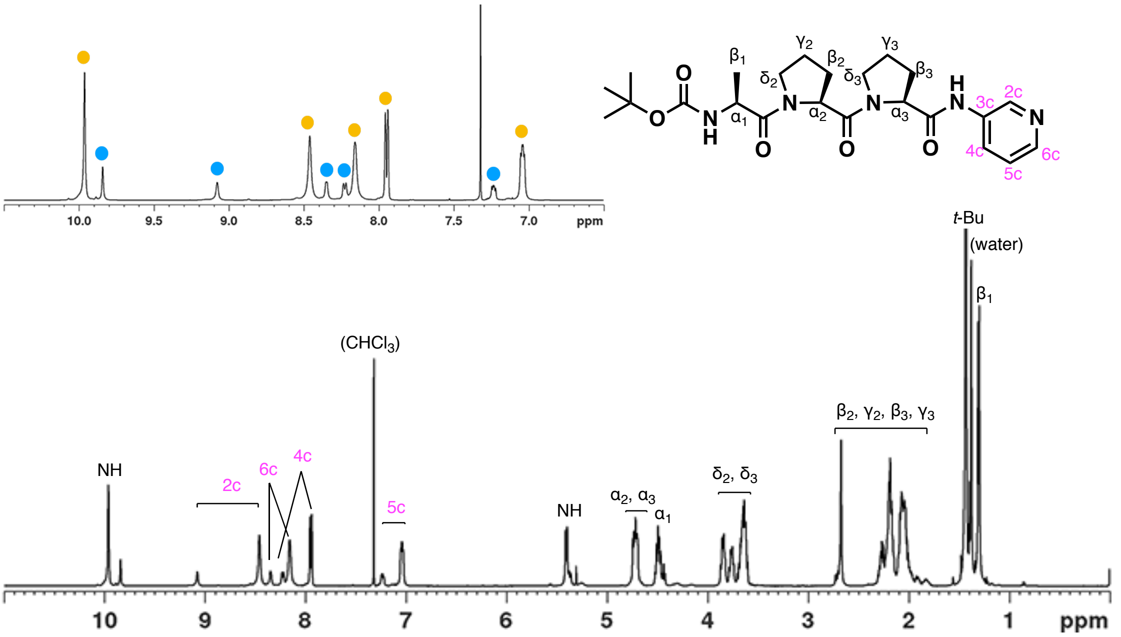

Supplementary Figure 32 | ^{1}H NMR spectrum (500 MHz, $CDCl_3$, 300 K) of **S3** (conformers exist).

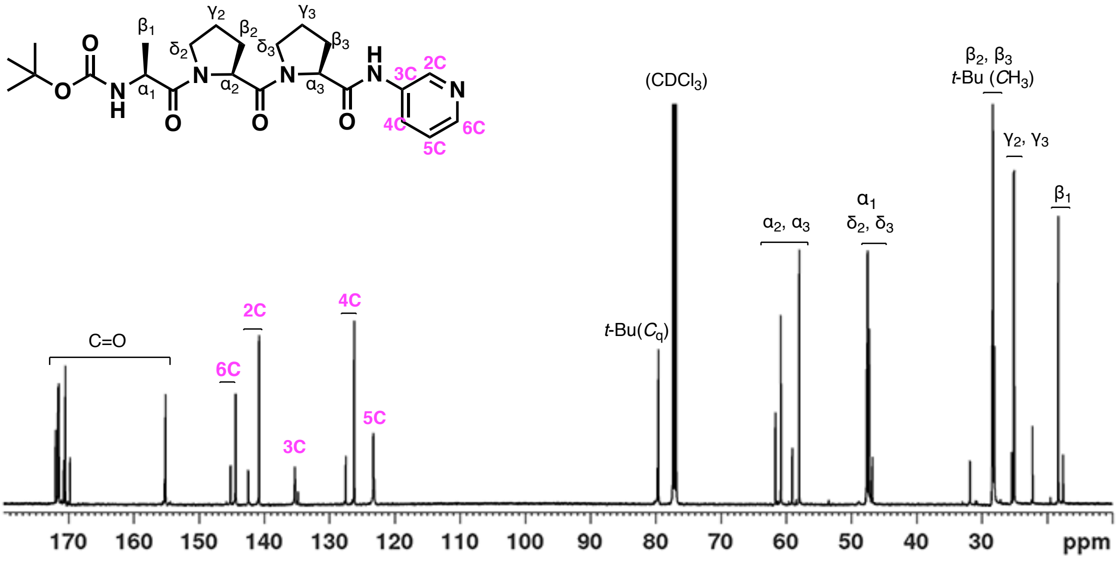

Supplementary Figure 33 | ^{13}C NMR spectrum (125 MHz, $CDCl_3$, 300 K) of **S3** (conformers exist).

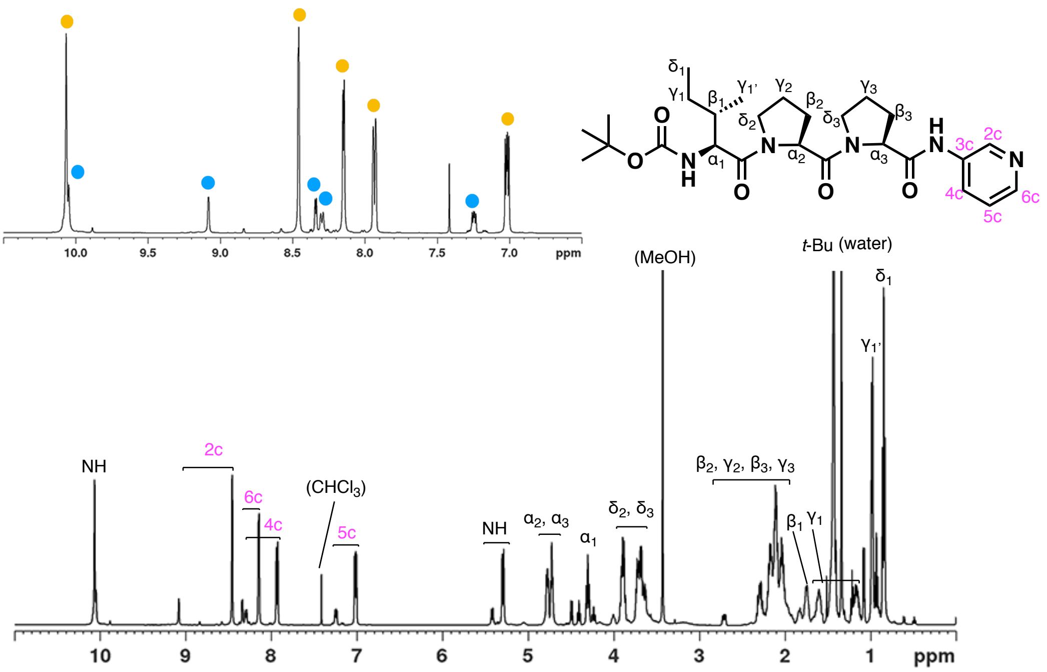

Supplementary Figure 34 | ^{1}H NMR spectrum (500 MHz, $CDCl_3$, 300 K) of **S4** (conformers exist).

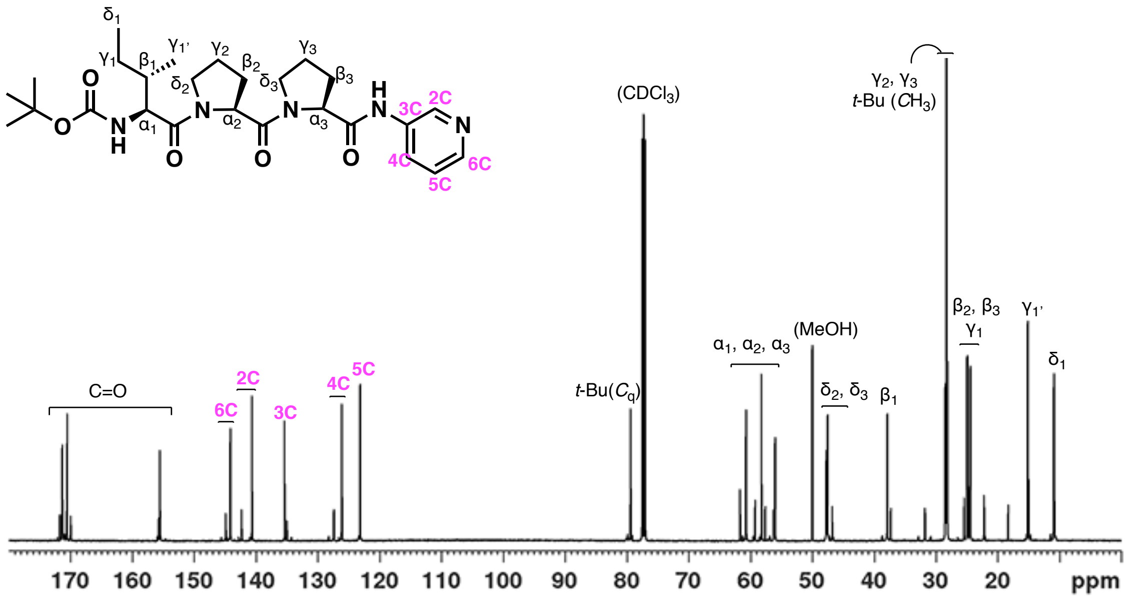

Supplementary Figure 35 | ^{13}C NMR spectrum (125 MHz, $CDCl_3$, 300 K) of **S4** (conformers exist).

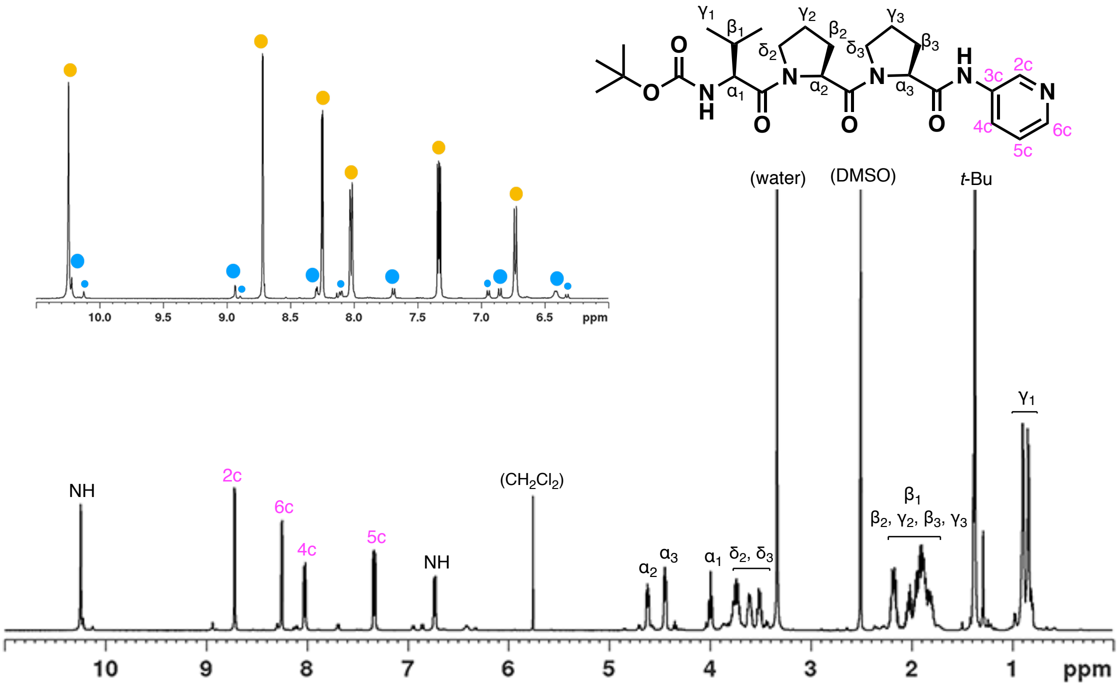

Supplementary Figure 36 | ^{1}H NMR spectrum (500 MHz, DMSO-d_6, 300 K) of **S5** (conformers exist).

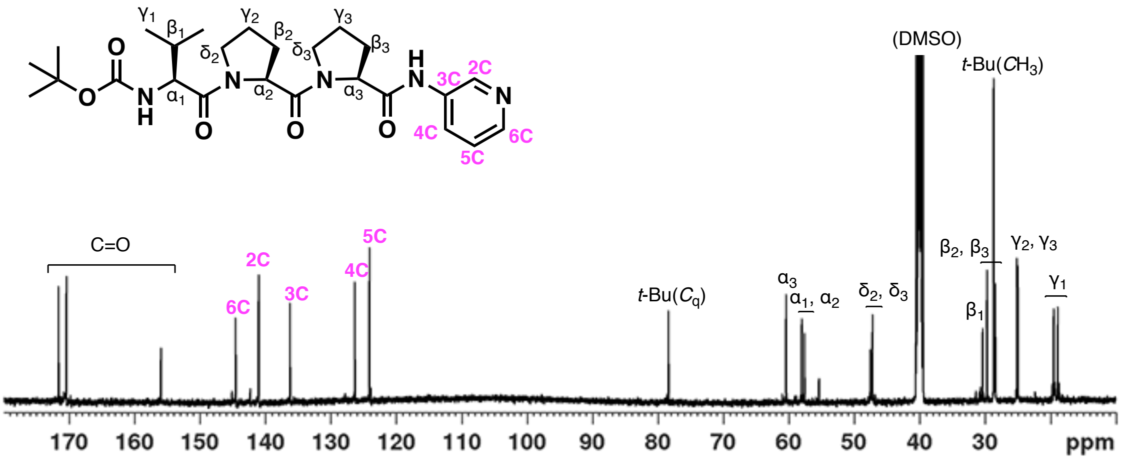

Supplementary Figure 37 | ^{13}C NMR spectrum (125 MHz, DMSO-d_6, 300 K) of **S5** (conformers exist).

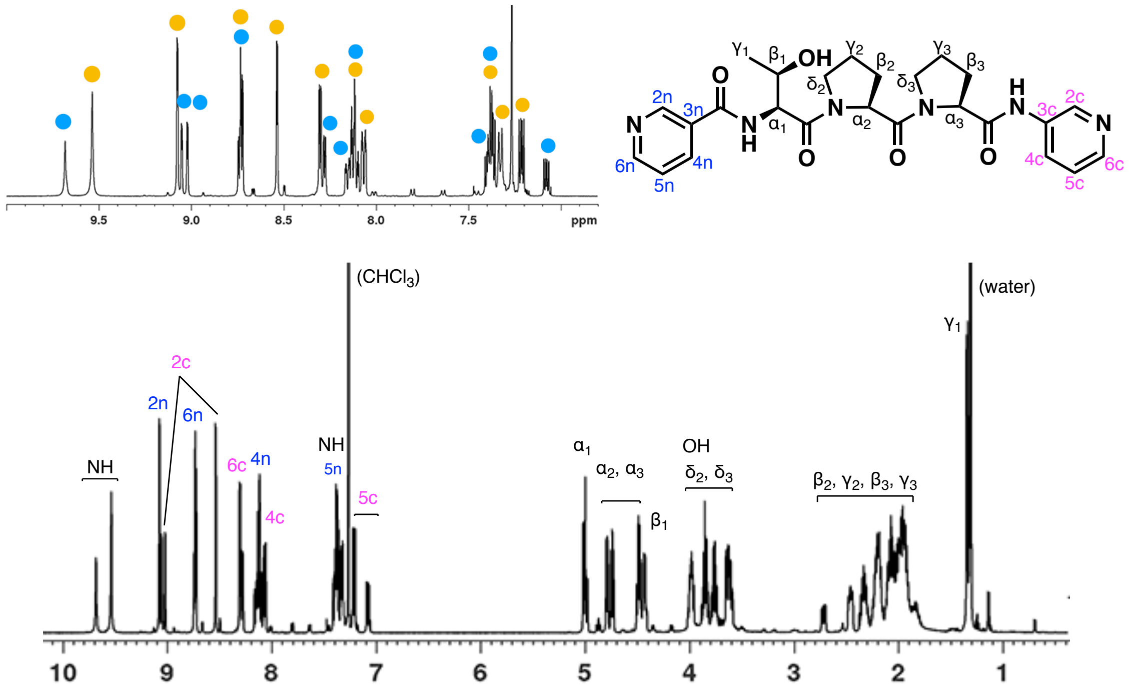

Supplementary Figure 38 | ^{1}H NMR spectrum (500 MHz, $CDCl_3$, 300 K) of **3** (conformers exist).

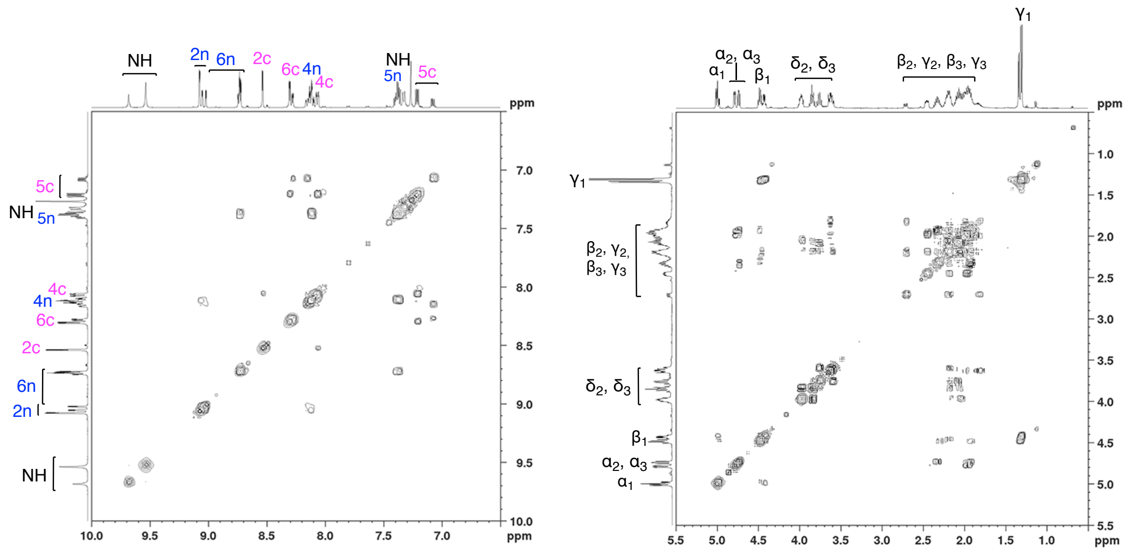

Supplementary Figure 39 | ^{1}H–^{1}H COSY spectra of **2** ($CDCl_3$, 300 K).

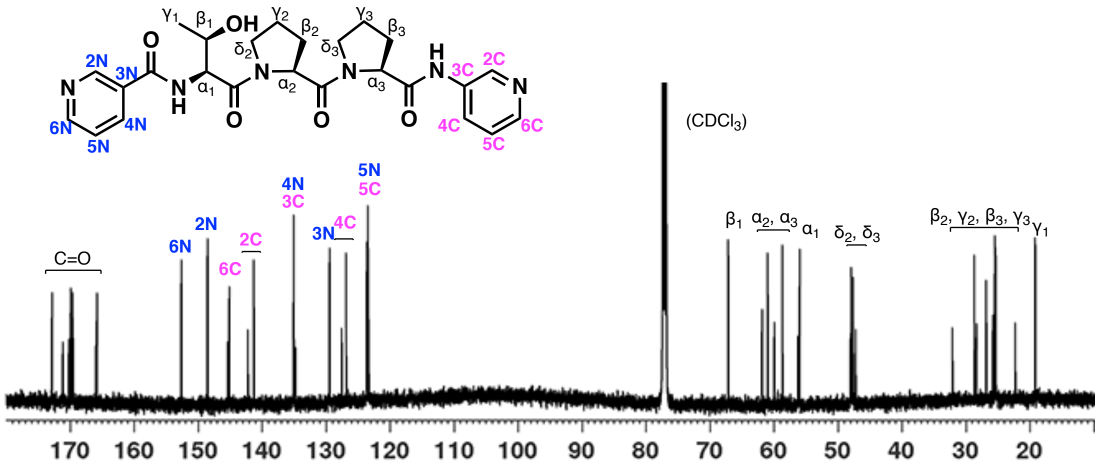

Supplementary Figure 40 | ^{13}C NMR spectrum (125 MHz, $CDCl_3$, 300 K) of **3** (conformers exist).

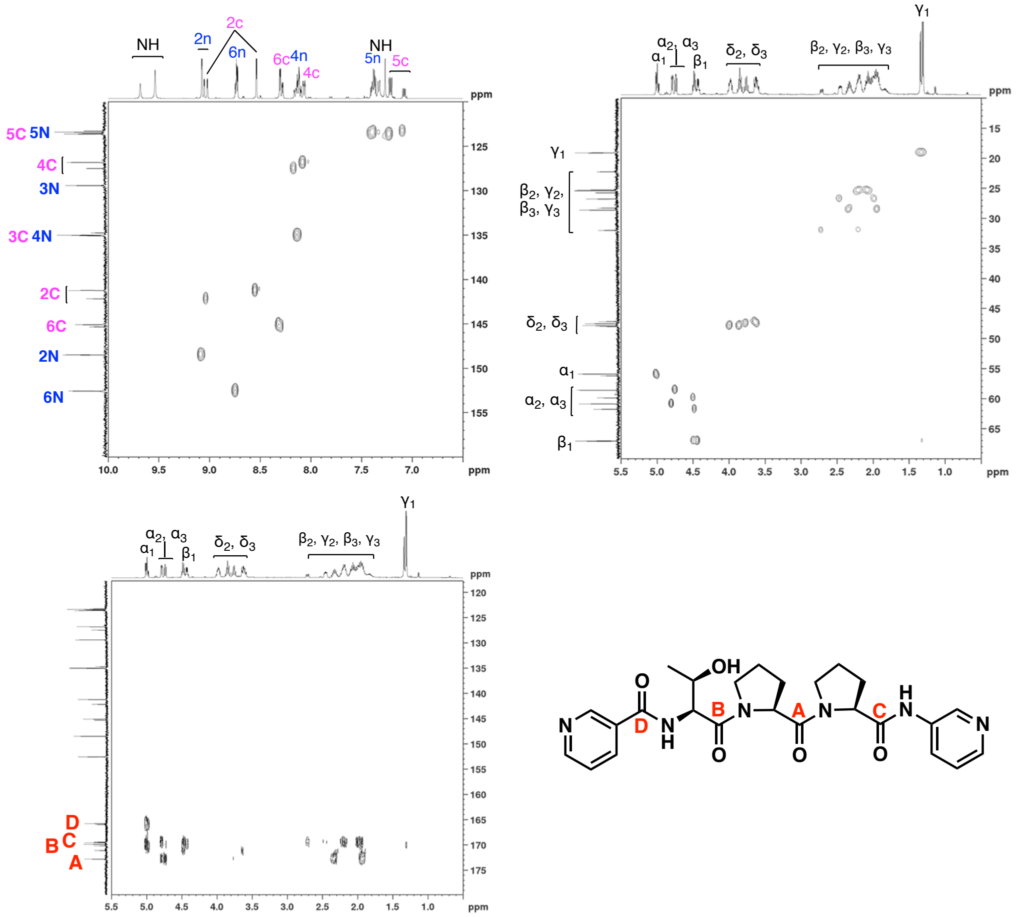

Supplementary Figure 41 | ^{1}H–^{13}C HSQC spectra (upper) and HMBC spectrum (bottom) of **3** ($CDCl_3$, 300 K).

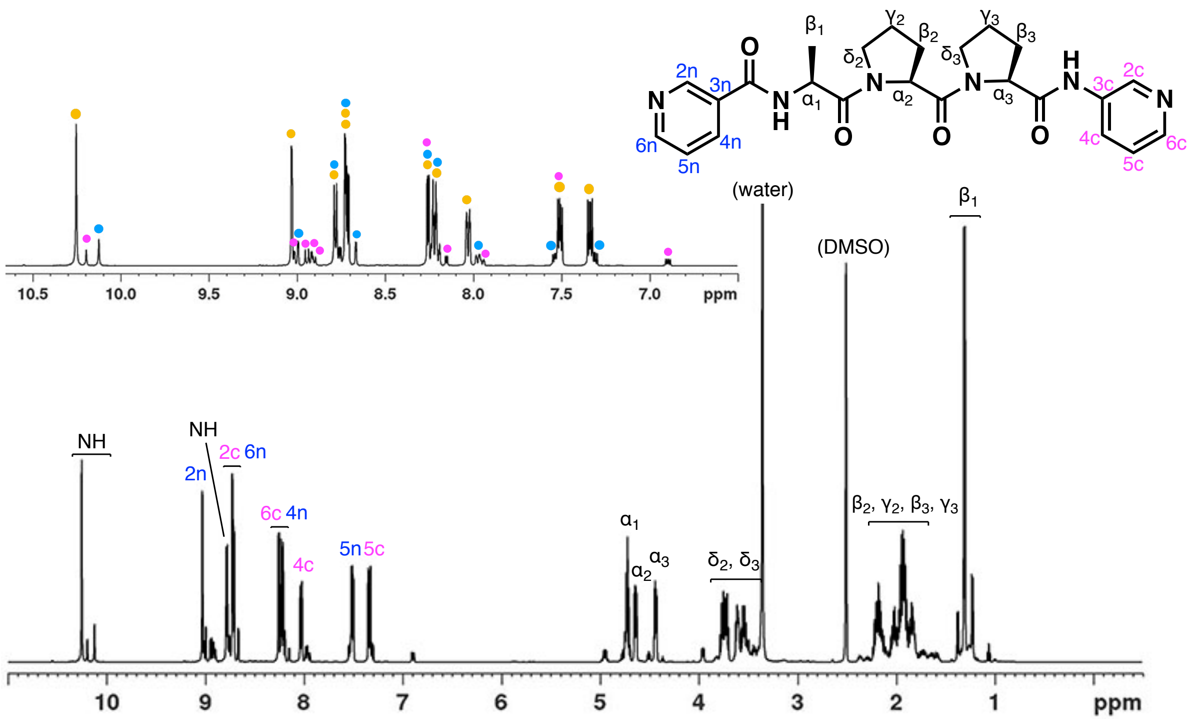

Supplementary Figure 42 | ^{1}H NMR spectrum (500 MHz, DMSO-d_6, 300 K) of **5** (conformers exist).

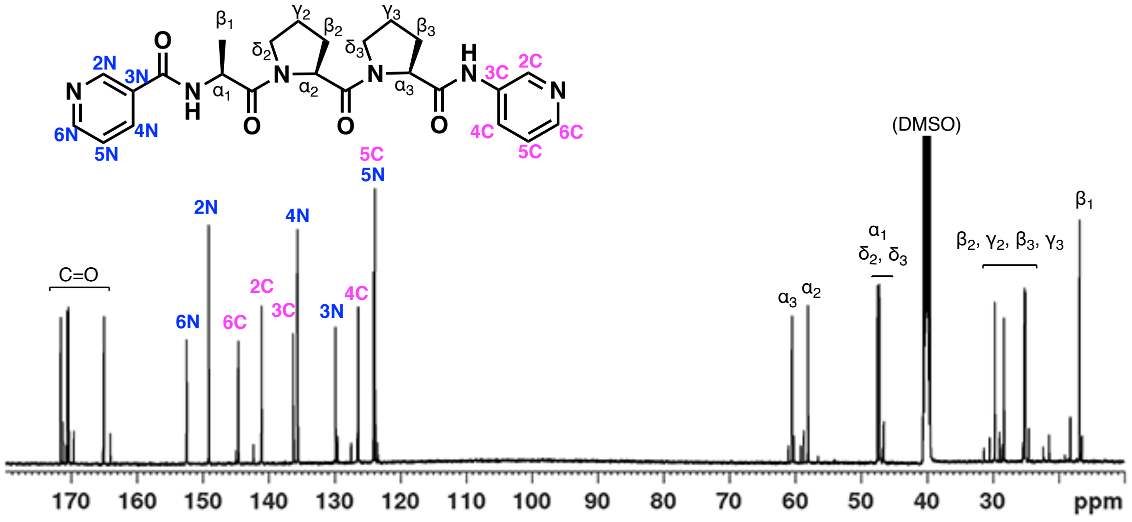

Supplementary Figure 43 | ^{13}C NMR spectrum (125 MHz, DMSO-d_6, 300 K) of **5** (conformers exist).

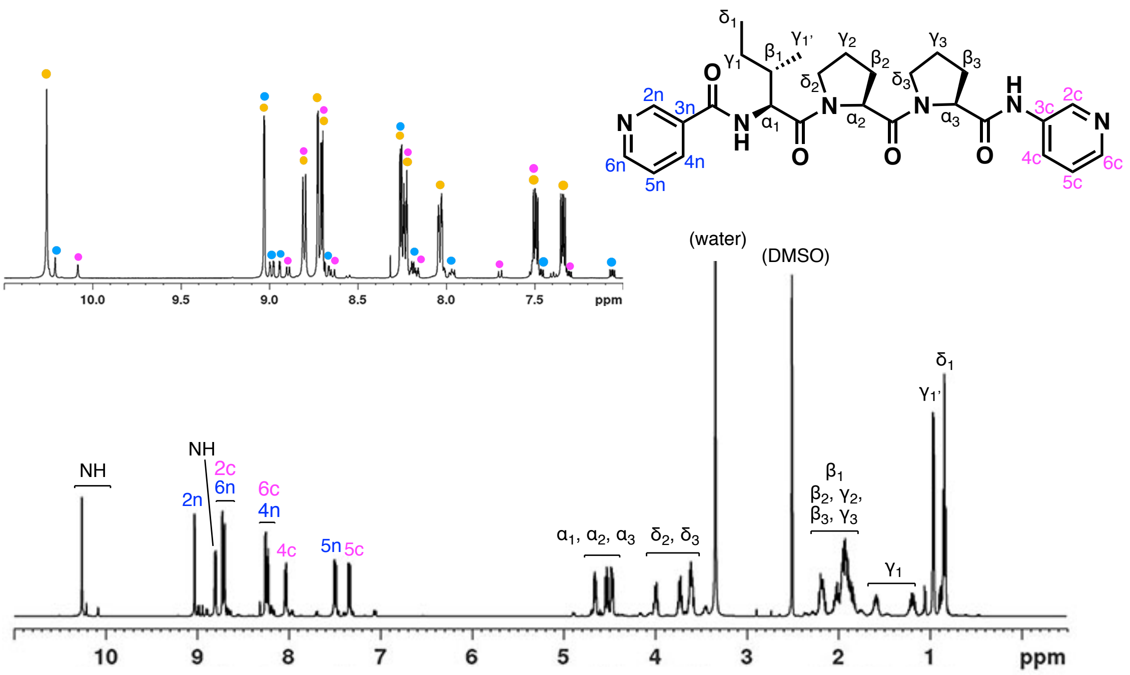

Supplementary Figure 44 | ^{1}H NMR spectrum (500 MHz, DMSO-d_6, 300 K) of **7** (conformers exist).

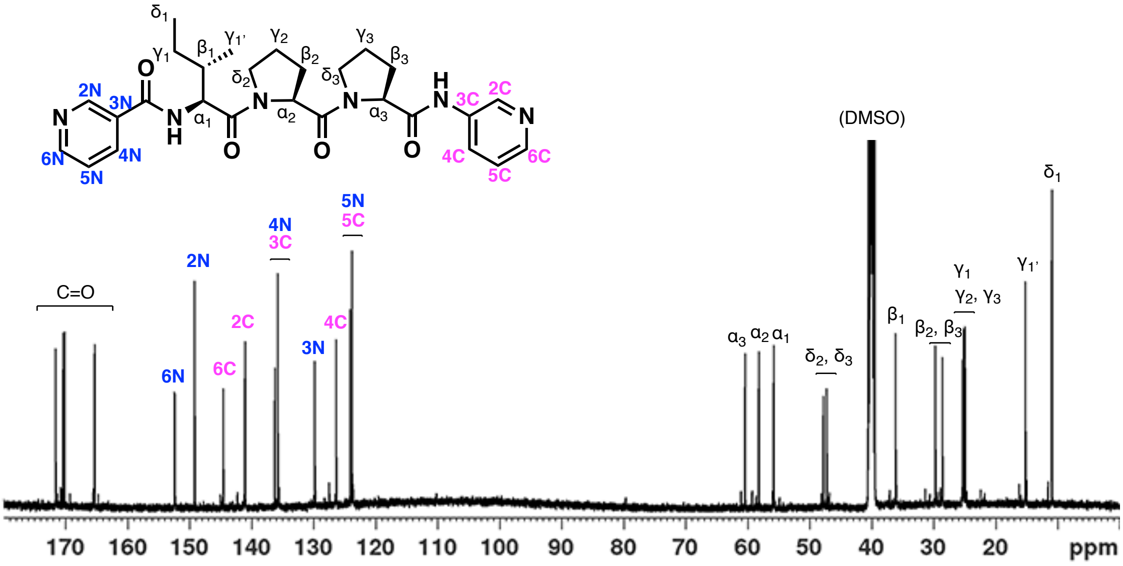

Supplementary Figure 45 | ^{13}C NMR spectrum (125 MHz, DMSO-d_6, 300 K) of **7** (conformers exist).

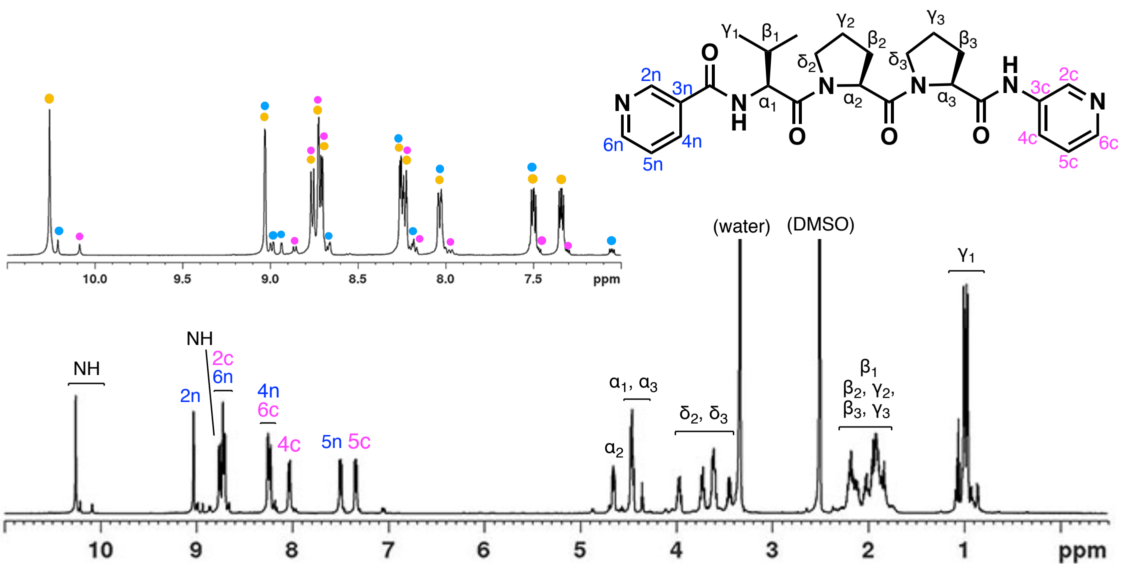

Supplementary Figure 46 | ^{1}H NMR spectrum (500 MHz, DMSO-d_6, 300 K) of **9** (conformers exist).

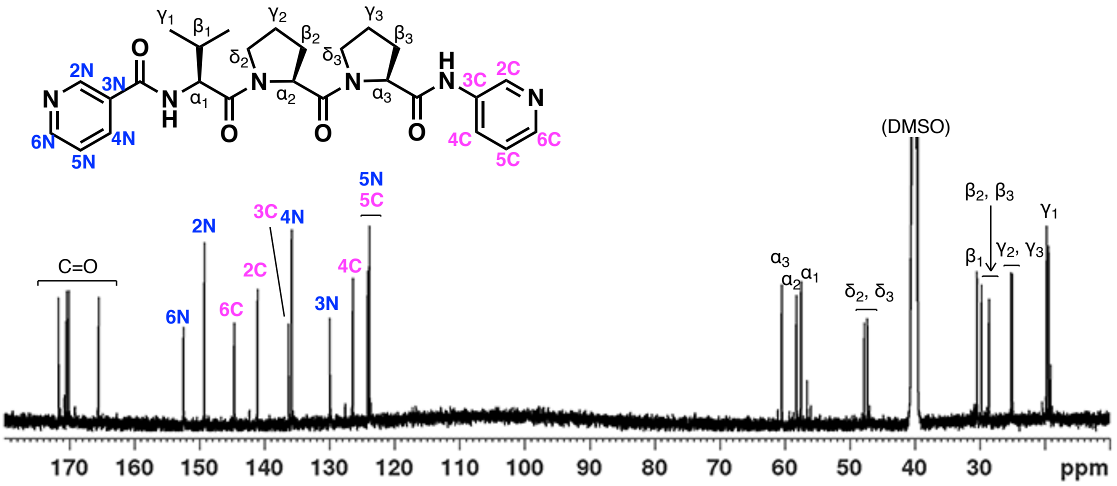

Supplementary Figure 47 | ^{13}C NMR spectrum (125 MHz, DMSO-d_6, 300 K) of **9** (conformers exist).

Supplementary Discussion

Topological analysis

The two topologies of $[4]_{12}$-cantenanes **2** and **4** (shown in Fig. 1b diagram) were analysed as topologically-different, which was confirmed by calculation of their link invariants shown below. These link invariants were calculated by using KNOT program.[3]

Link invariant of the topology of compound **2** (L_1):

crossing number

$$c(L_1) = 12$$

(total) linking number

$$lk(L_1) = -6$$

Conway polynomial

$$\nabla_{L1} = -16z^3$$

Jones polynomial

$$V_{L1} = -t^{\frac{3}{2}} + 3t^{\frac{5}{2}} - 6t^{\frac{7}{2}} + 10t^{\frac{9}{2}} - 15t^{\frac{11}{2}} + 17t^{\frac{13}{2}} - 20t^{\frac{15}{2}} + 17t^{\frac{17}{2}}$$
$$- 17t^{\frac{19}{2}} + 10t^{\frac{21}{2}} - 8t^{\frac{23}{2}} + 3t^{\frac{25}{2}} - t^{\frac{27}{2}}$$

HOMFLY polynomial

$$P_{L1}(v, z) = z^{-3}(-v^{-9} + 3v^{-11} - 3v^{-13} + v^{-15})$$
$$+z^{-1}(-6v^{-9} + 12v^{-11} - 6v^{-13})$$
$$+z(-4v^{-7} - 7v^{-9} + 11v^{-11})$$
$$+z^3(-v^{-3} - 3v^{-5} - 6v^{-7} - 6v^{-9})$$

Q-polynomial

$$Q(L_1) = 8x^{-3} - 12x^{-2} - 42x^{-1} + 47 + 140x - 92x^2 - 296x^3 + 68x^4 + 346x^5$$
$$+ 44x^6 - 190x^7 - 78x^8 + 30x^9 + 24x^{10} + 4x^{11}$$

Link invariant of the topology of compound **4** (L_2):

crossing number

$$c(L_2) = 12$$

(total) linking number

$$lk(L_2) = 6$$

Conway polynomial

$$\nabla_{L2} = 16z^5 + 16z^3$$

Jones polynomial

$$V_{L2} = -t^{-\frac{29}{2}} + 7t^{-\frac{27}{2}} - 20t^{-\frac{25}{2}} + 33t^{-\frac{23}{2}} - 50t^{-\frac{21}{2}} + 58t^{-\frac{19}{2}} - 63t^{-\frac{17}{2}} + 56t^{-\frac{15}{2}}$$
$$- 46t^{-\frac{13}{2}} + 29t^{-\frac{11}{2}} - 15t^{-\frac{9}{2}} + 5t^{-\frac{7}{2}} - t^{-\frac{5}{2}}$$

HOMFLY polynomial

$$P_{L2}(v, z) = z^{-3}(-v^{15} + 3v^{13} - 3v^{11} + v^9)$$
$$+z^{-1}(6v^{13} - 12v^{11} + 6v^9)$$
$$+z(4v^{13} - 23v^{11} + 19v^9)$$
$$+z^3(-v^{13} - 11v^{11} + 22v^9 + 6v^7)$$
$$+z^5(v^{11} + 9v^9 + 5v^7 + v^5)$$

Q-polynomial

$$Q(L_2) = 8x^{-3} - 12x^{-2} - 42x^{-1} + 47 + 76x - 60x^2 - 120x^3 + 60x^4 + 306x^5$$
$$+28x^6 - 382x^7 - 178x^8 + 134x^9 + 114x^{10} + 22x^{11}$$

The other topological descriptions can be available from the Knotilus website (http://knotilus.math.uwo.ca):

The archive code for the topology of $[4]_{12}$-cantenanes **2**: 12x-4-13

The archive code for the topology of $[4]_{12}$-cantenanes **4**: 12x-4-92

Supplementary References

1. Sawada, T., Matsumoto, A., & Fujita, M. Coordination-driven folding and assembly of a short peptide into a protein-like two-nanometer-sized channel. *Angew. Chem. Ed.* **2014**, *53*, 7228–7232.
2. Sawada, T., Yamagami, M., Ohara, K., Yamaguchi, K., & Fujita, M. Peptide [4]catenane by folding and assembly. *Angew. Chem. Ed.* **2016**, *55*, 4519–4522.
3. Kodama, K., KNOT program, http://www.math.kobe-u.ac.jp/HOME/kodama/knot.html (2004).
